# Conservation and *trans*-regulation of histone modification in the A and B subgenomes of polyploid wheat during domestication and ploidy transition

**DOI:** 10.1186/s12915-021-00985-7

**Published:** 2021-03-09

**Authors:** Zhenling Lv, Zijuan Li, Meiyue Wang, Fei Zhao, Wenjie Zhang, Changping Li, Lei Gong, Yijng Zhang, Annaliese S. Mason, Bao Liu

**Affiliations:** 1 Key Laboratory of Molecular Epigenetics of the Ministry of Education (MOE), Northeast Normal University, Changchun, 130024 China; 2 Department of Plant Breeding, Justus Liebig University, Heinrich-Buff-Ring 26-32, 35392 Giessen, Germany; 3 Department of Plant Breeding, University of Bonn, Katzenburgweg 5, 53115 Bonn, Germany; 4 National Key Laboratory of Plant Molecular Genetics, CAS Center for Excellence in Molecular Plant Sciences, Shanghai Institute of Plant Physiology and Ecology, Shanghai Institutes for Biological Sciences, Chinese Academy of Sciences, Shanghai, 200032 China; 5 University of the Chinese Academy of Sciences, Beijing, 100049 China

**Keywords:** Genome duplication, Ploidy transition, Histone modification, Gene expression, *trans*-regulation, Domestication, Wheat

## Abstract

**Background:**

Polyploidy has played a prominent role in the evolution of plants and many other eukaryotic lineages. However, how polyploid genomes adapt to the abrupt presence of two or more sets of chromosomes via genome regulation remains poorly understood. Here, we analyzed genome-wide histone modification and gene expression profiles in relation to domestication and ploidy transition in the A and B subgenomes of polyploid wheat.

**Results:**

We found that epigenetic modification patterns by two typical euchromatin histone markers, H3K4me3 and H3K27me3, for the great majority of homoeologous triad genes in A and B subgenomes were highly conserved between wild and domesticated tetraploid wheats and remained stable in the process of ploidy transitions from hexaploid to extracted tetraploid and then back to resynthesized hexaploid. However, a subset of genes was differentially modified during tetraploid and hexaploid wheat domestication and in response to ploidy transitions, and these genes were enriched for particular gene ontology (GO) terms. The extracted tetraploid wheat manifested higher overall histone modification levels than its hexaploid donor, and which were reversible and restored to normal levels in the resynthesized hexaploid. Further, while H3K4me3 marks were distally distributed along each chromosome and significantly correlated with subgenome expression as expected, H3K27me3 marks showed only a weak distal bias and did not show a significant correlation with gene expression.

**Conclusions:**

Our results reveal overall high stability of histone modification patterns in the A and B subgenomes of polyploid wheat during domestication and in the process of ploidy transitions. However, modification levels of a subset of functionally relevant genes in the A and B genomes were *trans*-regulated by the D genome in hexaploid wheat.

**Supplementary Information:**

The online version contains supplementary material available at 10.1186/s12915-021-00985-7.

## Background

Polyploidy is a pervasive driving force in evolution, particularly in higher plants [1]. Polyploids can form when either a hybridization or somatic chromosome doubling event occurs and results in individuals that contain three or more sets of chromosomes from two or more different species (allopolyploids), or from a single species (autopolyploids). Polyploids are common in plants, with 30–80% of extant species are recent polyploids (neopolyploids), while historic polyploidization events occurred in virtually all ancestral species lineages [2–6]. Polyploids are also over-represented in crop species, indicating they have been more favorably domesticated than their diploid progenitors [7–10]. However, despite prevalence of polyploids and an enduring interest by scientists in the causes and consequences of whole genome duplication (WGD), there are many unanswered questions about how polyploids form, stabilize and establish as evolutionarily successful entities, even less is known about the genetic and genomic bases underlying these processes.

An immediate challenge facing newly formed allopolyploids is genome regulation. After allopolyploid formation, cells contain two or more sets of abruptly merged, potentially competing genetic instructions and conflicting regulatory networks [6, 11–13], as well as cellular and physiological constraints due to the presence of twice as much DNA or more [13]. One major mechanism by which nascent polyploid plants can overcome these incompatibilities or conflicts and restore normal growth and development is via rapid changes in epigenetic regulation of gene expression and genome re-stabilization [14–16]. Indeed, an array of investigations in diverse plants have documented that allopolyploidization induces a cascade of rapid epigenetic modifications such as altered DNA methylation via differential titration of non-coding RNAs [17–21]. Analyses of homoeologous DNA methylation pattern in hexaploid wheat suggested that tri-genome methylation is significantly more conserved across the accessions compared to uni- and bi-genome methylation [22, 23]. Beside DNA methylation, histone modifications also play important roles in polyploid formation and stabilization [21, 24]. Notwithstanding these studies, to date the interplay of subgenome autonomy vs. *trans*-genome interaction associated with histone modifications in a given allopolyploid following allopolyploidization or during the course of evolution and domestication remains poorly understood.

As one of the most important food crops world-wide, common or bread wheat (*Triticum aestivum* L.) is also a textbook example of speciation via allopolyploidization, and hence, an excellent model to explore processes of genome regulation post-polyploidization and in the course of evolution and domestication. Bread wheat is a young allohexaploid species, formed less than 10,000 years ago via allopolyploidization between a primitively domesticated tetraploid wheat (*T. turgidum*, ssp. *dicoccum* or *durum*, genome AABB, 2*n* = 28) and goat grass *Aegilops tauschii* (genome DD, 2*n* = 14), most likely as a result of selection during human agriculture [25]. The three subgenomes present in bread wheat diverged approximately 6.5 million years ago, while tetraploid wheat (AABB) is thought to have arisen approximately 0.5–0.8 million years ago [25]. Important for studies of allopolyploid evolution, it is possible to recreate the ancestral hybridization events that led to allohexaploid bread wheat speciation by hybridizing tetraploid wheat and goat grass followed by induced chromosome doubling, and to recapitulate gene expression regulation and genome modification patterns that may have occurred in the early generations of this speciation process. Previous research has also resulted in the production of a unique type of “extracted tetraploid” that contains only the A and B genomes of bread wheat [26, 27]. The availability of this unique material as well as the rapid accumulation of genetic and genomic resources for polyploid wheat and its progenitor species allow the investigation of several pressing questions with respect to how allopolyploids regulate gene expression by epigenetic modifications, as well as the potential effects of domestication and subgenome co-evolution on genome and epigenome dynamics.

It is also conceivable that successful formation of hexaploid wheat is contingent on properties of tetraploid wheat which itself entails stabilization and coordination of its two (A and B) constituent subgenomes. In this study, by employing a set of tetraploid and hexaploid wheat lines we analyzed the stability and dynamics of two typical euchromatin histone modification markers (H3K4me3 and H3K27me3) in relation to allopolyploid formation, domestication and ploidy level transition, and assessed their attendant impacts on subgenome gene expression. We found the A-subgenome in all three tetraploid wheats showed higher levels of modification of both histone markers than the B-subgenome except for extracted tetraploid wheat (ETW) of H3K27me3, and the evolution and domestication at hexaploid level had a distinct impact on the epigenetic modifications between the subgenomes compared to these processes at the tetraploid level. We also found that 76.7–96.1% triad homoeologous genes maintained stable modification levels while 3.9–23.3% triads genes showed variable levels in the ploidy transition processes, suggesting predominant autonomy in modification of the AABB subgenomes accompanied with moderate *trans*-regulatory effects by the DD subgenome in hexaploid wheat. These results may partially explain, from an epigenetic perspective, why ETW as an independent AABB component albeit can be extracted from hexaploid wheat to form a viable and reproducing plant but manifests severe phenotypic defects that notwithstanding can be rescued in resynthesized hexaploid XX329 [26, 27]. Together, our results provide new insights regarding the highly orchestrated stability and dynamics of chromatin epigenetic modifications and their biological relevance in the course of evolution and domestication of both tetraploid and hexaploid wheat.

## Results

### Quality assessment of the ChIP-seq data

**Fig. 1:**
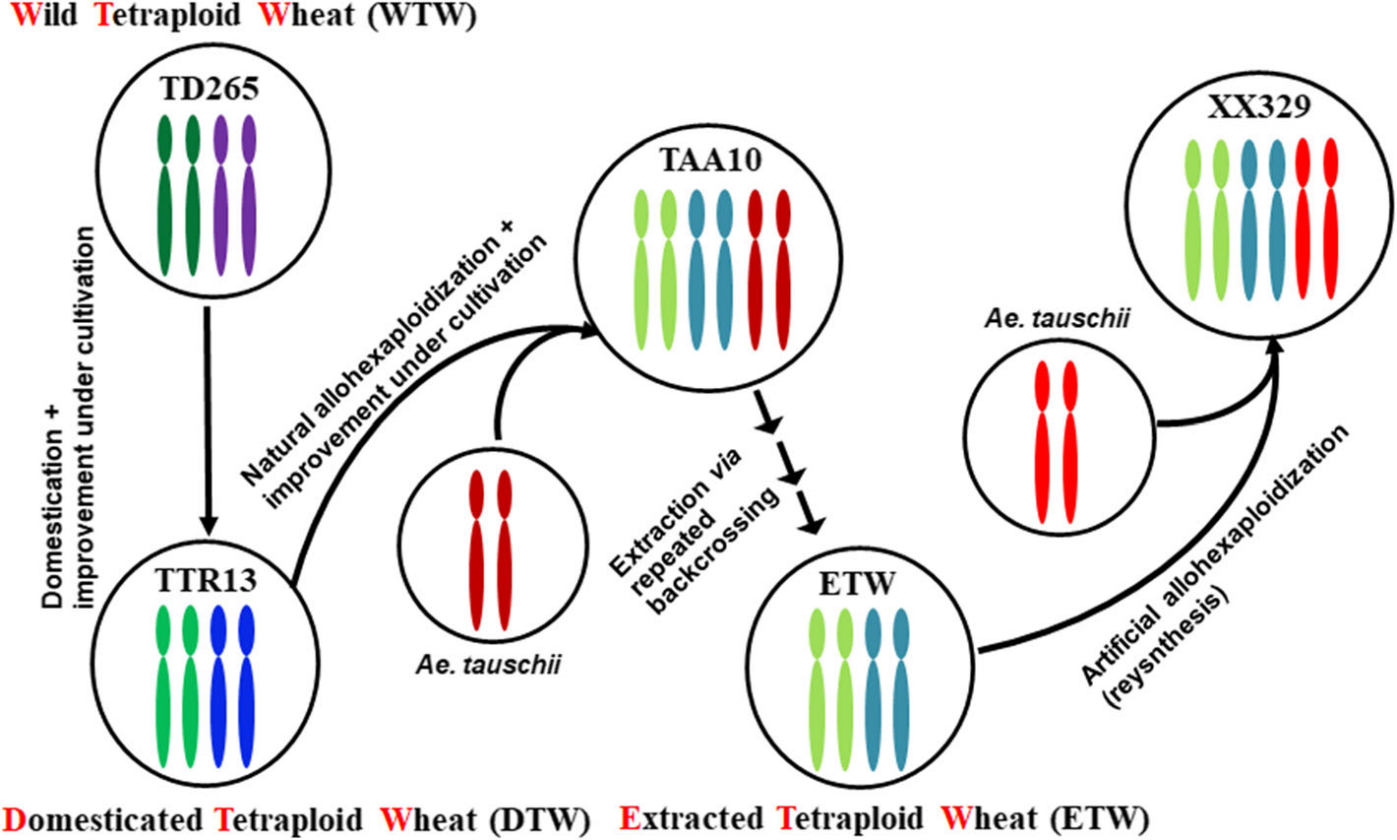
Relationships between the wheat lines used in this study. Different colored diagrammed chromosomes show the putative genetic and evolutionary relationships between the lines

First, we evaluated quality of our ChIP-seq data of all studied wheat lines by checking the sequence alignments after mapping the raw data to the subgenomes of tetraploid and hexaploid wheat using the hexaploid common wheat (cv. CS) reference genome (RefSeqv1.0, [28]). The genetic relationships among the wheat lines studied are diagrammatically depicted (Fig. 1). We chose only uniquely mapped reads for analyses. For tetraploids and hexaploids (Fig. 1), the reads were quantified in a homoeolog-specific manner. Thus, in theory the reads should map to the A and B subgenomes in a tetraploid wheat, and A, B, and D subgenomes in a hexaploid wheat, in more or less equal proportions, while reads of the D-genome diploid (*Aegilops tauschii*) should map to the D genome only. Indeed, we found our ChIP-seq reads fully accorded with these expectations (Additional file 1: Figure S1). Next, we quantified counts of H3K4me3 and H3K27me3 in all plant lines studied (Fig. 1) and tested correlations between the two biological replicates of each line. We found the correlations between the two biological replicates were high, with average coefficients of 0.99 and 0.91 for H3K4me3 and H3K27me3, respectively (Additional file 1: Figure S2). To further interrogate the accuracy of our data quality estimation, we separated the A- and B-subgenomes or considered AABB genomes as a whole to calculate the correlations between the biological replicates or between different plant lines, and we found the high correlations were robustly supported (Additional file 1: Figure S2). We also assessed the quality of our ChIP-seq data by comparing them with the previously published ChIP-seq data for the hexaploid common wheat reference genotype Chinese Spring (CS) [28]; we found a high consistency between the two data sets (Additional file 1: Figure S2). Taken together, our ChIP-seq data were of sufficiently high quality for further analyses.

### The A and B subgenomes of tetraploid wheat are asymmetrically modified by H3K4me3 and H3K27me3

**Fig. 2:**
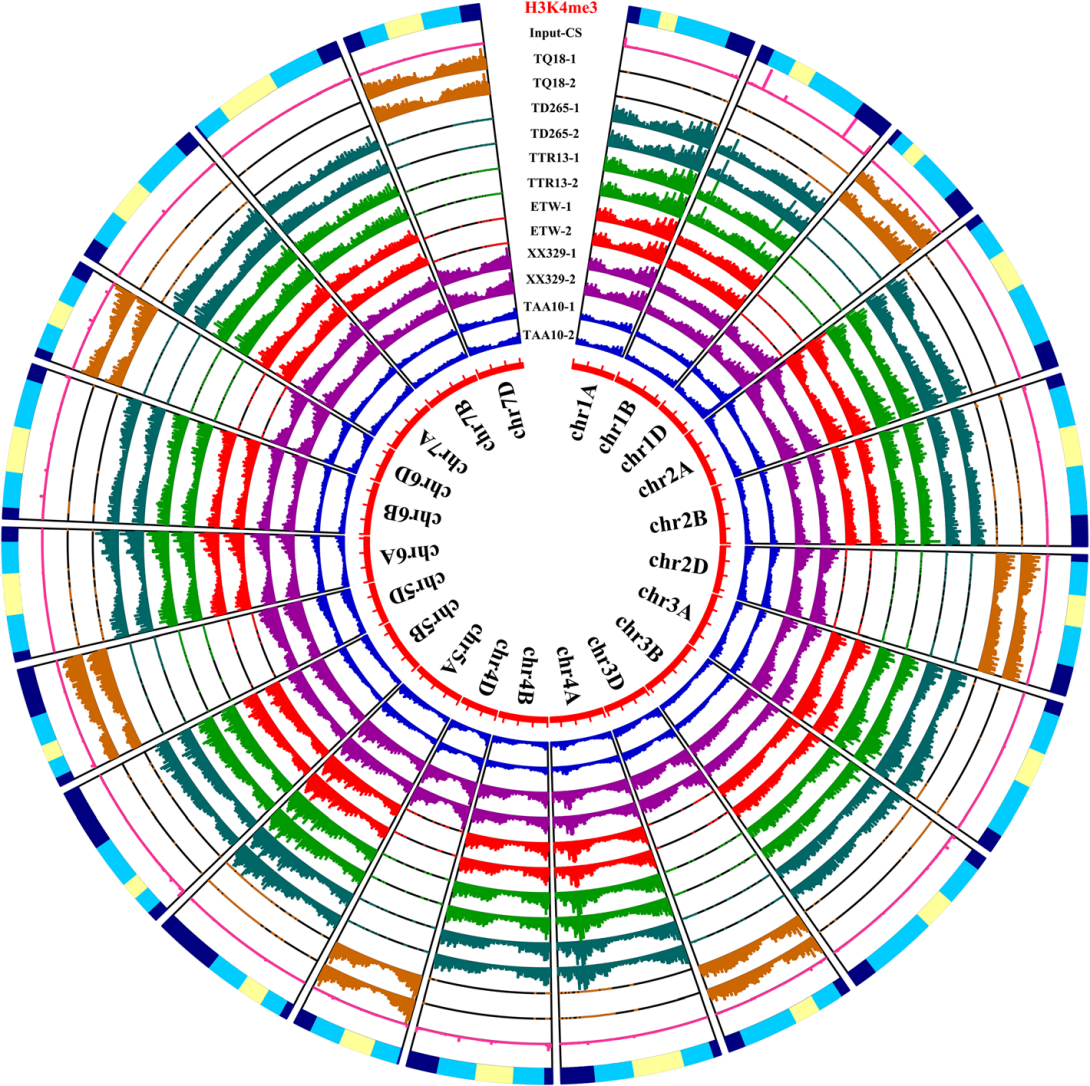
Genome and chromosome distribution of H3K4me3 in all the wheat lines used in this study. From the outside to the inside, the lines are DD diploid “TQ18,” tetraploid wheats “TD265” (wild), “TTR13” (domesticated), and “ETW” (extracted from hexaploid wheat), hexaploid wheats “XX329” (resynthesized) and “TAA10” (donor to ETW). Different colors represent different lines, each with two biological replicates. The outermost layer is the position information of chromosomes, in which pale yellow denotes the centromeric and pericentromeric region (Proximal (C)), light blue represents the chromosome arm (Interstitial (R2a/R2b)), and dark blue indicates the end of the chromosome (Distal (R1/R3)). The punctuated innermost red lines represent length of all the seven wheat chromosomes [28]

We found that both H3K4me3 and H3K27me3 markers exhibited genome-wide distribution in each tetraploid wheat (Fig. 2; Additional file 1: Figure S3). At the chromosome level, H3K4me3 was quantitatively enriched in the following descending order: distal > interstitial > proximal along each chromosome (Fig. 2), consistent with previous findings in hexaploid common wheat. Unexpectedly, we found reads of H3K27me3 were predominantly aggregated to the distal extremity of each chromosome (Fig. 1; Additional file 1: Figure S3). Also, we noted that the overall abundance of H3K27me3 was substantially lower than H3K4me3 (Fig. 1; Additional file 1: Figure S3).

To exclude confounding factors and ambiguities, we only focused on triad genes in this study, which were previously identified and defined for hexaploid wheat, cv. Chinese Spring (CS) [28]. The triad genes show a 1:1:1 correspondence across the three homeologous subgenomes, and include17,753 genes in total. To quantify the relative histone modification level of each homoeolog across each of the triads, we normalized the TPM (Transcripts Per Kilobase Million) reads for each gene within a given triad. We defined a triad as modified by a histone marker when the A or B subgenome homeolog was > 0.5 TPM [28]. Using this criterion, we defined 14,448 (81.4% of 17,753) triads as H3K4me3-modified, and 8960 (50.5% of 17,753) triads as H3K27me3-modified in at least one of the three tetraploid wheat lines (Additional file 3: Table S2). This information allowed quantitative assessment of whether the A and B homeologs in a given triad were equally modified by each of the histone markers or whether there was biased modification for one homeolog, and enabled tabulation of the triad gene numbers corresponding to the A = B, A > B and A < B patterns, respectively (Additional file 3: Table S2).

**Fig. 3:**
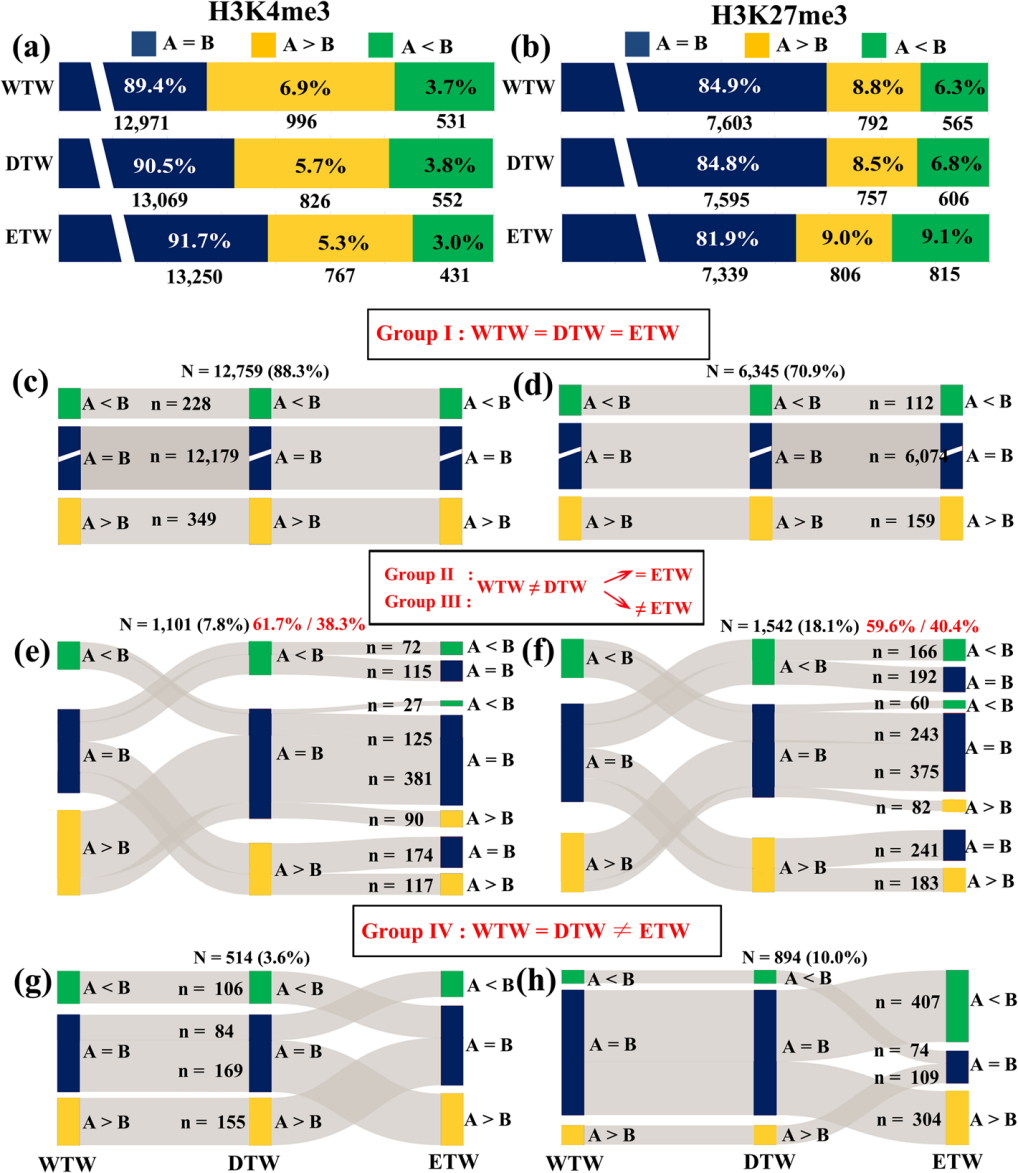
Histone modification pattern differences between subgenomes in wild, domesticated and extracted tetraploid wheats. Histone modification patterns of A = B, A > B and A < B between the A and B subgenomes in the three tetraploid wheats: wild tetraploid wheat (WTW, accession TD265), domesticated tetraploid wheat (ETW, cv. TTR13) and extracted-tetraploid wheat (ETW). Relative proportions of the three patterns (A = B, A > B, A < B) of the two markers, H3K4me3 (**a**) and H3K27me3 (**b**), in the three tetraploid wheats were presented. Tracking of pattern changes in the process of WTW → ETW → ETW for the two markers, H3K4me3 (**c**, **e**, **g**) and H3K27me3 (**d**, **f**, **h**), were illustrated

We found that 1527 (10.6%), 1378 (9.5%), and 1198 (8.3%) triad genes showed biased modification by H3K4me3, while 1357 (15.1%), 1363 (15.2%), and 1621 (18.1%) showed biased modification by H3K27me3 between the A and B subgenomes in wild tetraploid wheat (WTW, accession TD265), domesticated tetraploid wheat (DTW, cv. TTR13) and extracted tetraploid wheat (ETW), respectively (Fig. 1; Fig. 3a, b). Strikingly, subgenome A in all three tetraploid wheat lines showed higher levels of histone methylation for both markers than subgenome B (*p* values < 0.001, Binom test) except in ETW by H3K27me3 (*p* value = 0.843, Binom test). This indicates methylation levels in both markers were asymmetric between the A and B subgenomes in natural wild and domesticated tetraploid wheats. These results mirror a previous study of transcriptome analysis, which showed subgenome A manifesting an overall higher expression than subgenome B in both wild and domesticated tetraploid wheat of different tissues, suggesting the global scale (i.e., subgenome-wide) histone methylation and gene expression were likely coupled [29].

We quantified relative proportions of the three patterns for each marker between the A and B subgenomes (A = B, A > B or A < B) in each of the three tetraploid wheat lines. We found that the relative proportions of the three patterns were highly conserved among the three tetraploid wheat lines for both markers except in ETW for H3K27me3 (Fig. 3a, b). Specifically, in all three lines A = B was the predominant pattern for both markers (89.4–91.7% for H3K4me3, and 81.9–84.9% for H3K27me3), followed by the A > B pattern in all three lines (5.5–6.9% for H3K4me3, and 8.5–9.0% for H3K27me3), and with A < B being the least in two lines, WTW (TD265) and DWT (TTR13) (3.0 and 3.7% for H3K4me3, and 6.3 and 9.1% for H3K27me3, respectively) and at the same proportion as the A > B pattern in ETW (Fig. 3a, b). In all cases except for A > B and A < B in ETW, the between-pattern differences were statistically significant in each line (Additional file 4: Table S3, *p* values < 0.05, prop.test). Together, these results suggest that although the histone methylation of A and B subgenomes by the two typical euchromatin histone markers is generally conserved in both tetraploid and hexaploid wheats, further modification to some of the triad genes has occurred in hexaploid wheat.

### Effects of domestication and evolution under cultivation on subgenome histone methylation

To explore whether the relative histone methylation levels to the A and B subgenomes have been impacted by domestication and evolution under cultivation at the tetraploid and hexaploid levels, we analyzed how the modification states of A vs. B homologs of each of the triad genes have changed from wild tetraploid wheat (WTW, accession TD265) to domesticated tetraploid wheat (DTW, cv. TTR13), and then to the extracted A and B genomes of domesticated hexaploid common wheat (ETW) (Fig. 1). We categorized the methylation-modified triad genes into four groups. Group I is triad genes with pattern conservation, that is, the A vs. B methylation patterns of H3K4me3 or H3K27me3 remained the same (statistically equal) across the three tetraploid wheat lines (methylation pattern: WTW = DTW = ETW). In other words, the relative histone methylation levels of A vs. B of these triad genes were stable throughout evolution, domestication, and evolution under cultivation at both tetraploid and hexaploid levels. Groups II and III refer to triad genes in which the A vs. B methylation patterns were statistically different between WTW and DTW (methylation pattern: WTW ≠ DTW). Thus, if these modification-altered triad genes remained invariable in ETW, then, they were classified into Group II, while those which showed further changes in ETW were classified into Group III. Group IV refers to triad genes in which the A vs. B methylation patterns were conserved between WTW and DTW but both were statistically different from ETW (methylation pattern: WTW = DTW ≠ ETW) (Additional file 5: Table S4).

**Fig. 4:**
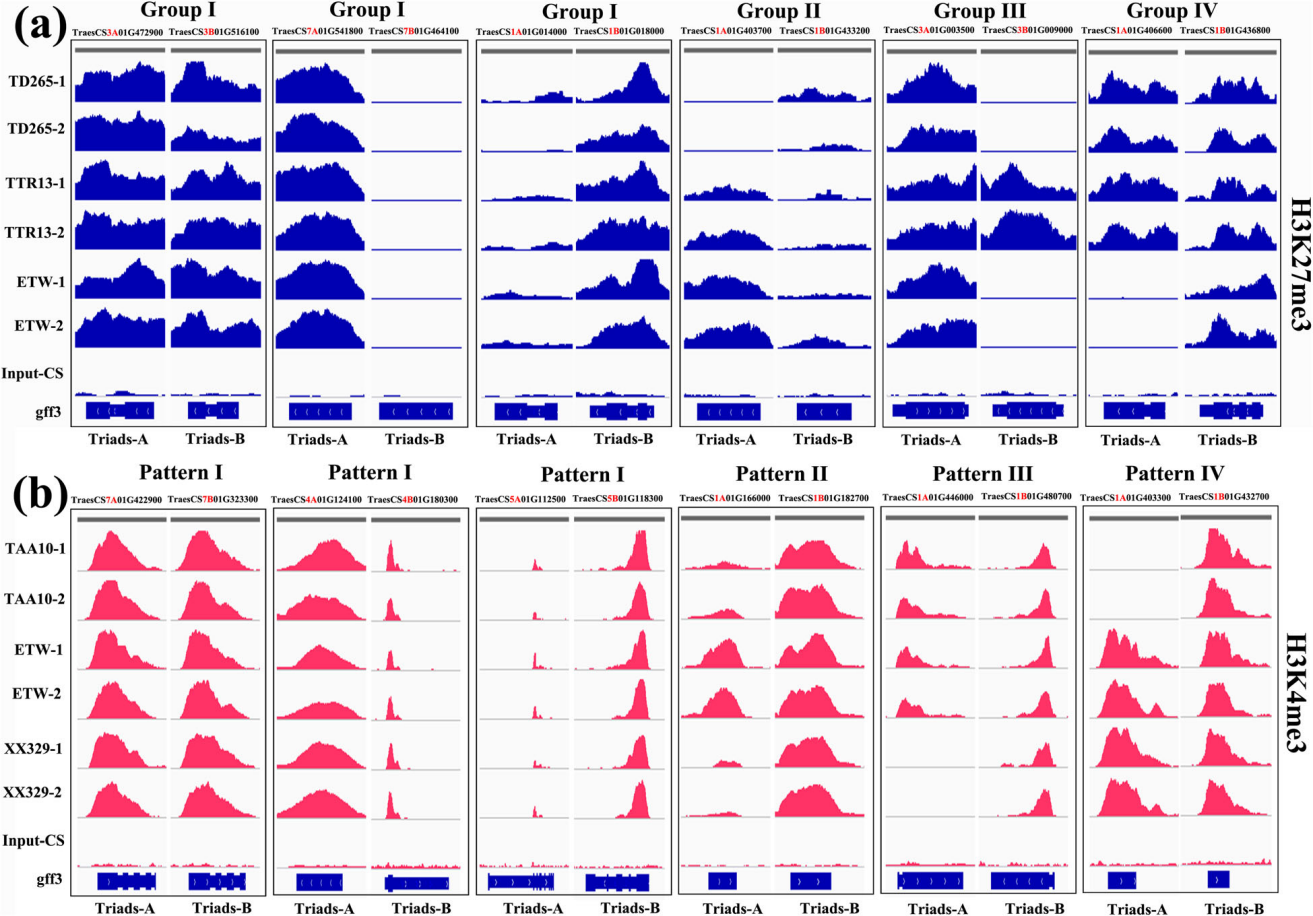
Examples of conservation and dynamics of the two histone modification markers, H3K4me3 and H3K27me3, exemplified by integrative genomics viewer (IGV) snapshots. **a** Groups I–IV histone modification patterns of H3K27me3 in the WTW → DTW → ETW process. For Group I (conserved), the three possible relationships, namely, A = B, A < B and A > B, in each of the WTW → DTW → ETW steps are presented; Group II shows changes that occurred in DTW and maintained in ETW; Group III shows changes that occurred in DTW and changed further in ETW; Group IV shows changes that only occurred in ETW. **b** Groups I–IV histone modification patterns of H3K4me3 in the TAA10 → ETW → XX329 ploidy transition process. For Pattern I (conserved), all three changing patterns, namely, A = B, A < B and A > B, in each of the TAA10 → ETW → XX329 steps are presented; Pattern II indicates reversible changes that are exclusively dependent on the presence of DD subgenome; Pattern III indicates changes that only occurred in XX329, reflecting prompt *trans*-subgenome regulation mediated by the presence of a novel DD genome (newly introduced from *Aegilops tauchii*); Pattern IV indicates changes that occurred in ETW and XX329, suggesting possible distinct functions of the AABB subgenome mediated by co-existence with the DD genome

The proportions of group I for H3K4me3 and H3K27me3 were 88.3% and 70.9%, respectively (Additional file 5: Table S4; Fig. 3c, d), indicating the relative levels of H3K4me3 between the A and B subgenomes was conserved to a greater extent than H3K27me3 (prop.test *P* < 0.05) in the process of domestication and evolution under cultivation at both tetraploid and hexaploid levels. The proportions of group II and group III together for H3K4me3 and H3K27me3 were 7.8% and 18.1%, respectively (Additional file 5: Table S4; Fig. 3e, f). Of these, the majority (61.7% for H3K4me3 and 59.6% for H3K27me3) retained the same A vs. B patterns between WTW and ETW (i.e., group II), suggesting no further change in either marker at the hexaploid level for this set of triad genes; the A vs. B patterns of the rest (38.3% for H3K4me3 and 40.4% for H3K27me3) were different between WTW and ETW (i.e., group III), indicating these triad genes have undergone additional histone methylation changes at the hexaploid level (Additional file 5: Table S4; Fig. 3e, f). The proportions of group IV for H3K4me3 and H3K27me3 were 3.6% and 10%, respectively (Fig. 3g, h; Additional file 5: Table S4), which reflect specific changes of histone methylation in hexaploid wheat. We showcased a subset of representative modification-enriched genomic regions for each of the histone markers (H3K4me3 and H3K27me3), which represented all possible modification patterns as integrative genomics viewer (IGV) snapshots during the WTW → DTW → ETW process (Fig. 4a; Additional file 1: Figure S4a).

To explore whether the triad genes manifesting different patterns of histone methylation were functionally relevant, we performed Gene Ontology (GO) enrichment analyses for each group. We found that the GO category enrichments were distinct between the two markers but largely similar among the triad gene groups for a given marker. For example, the enriched GO terms by the three changed groups of H3K4me3-modified triad genes were involved in transcription regulation, enzyme activities, growth factors, and different metabolic pathways, while H3K27me3-modified triad genes were enriched for responses to auxin and desiccation, nucleotide binding and kinase activity (Additional file 1: Figure S5a, S6a; Additional file 6: Table S5; Additional file 7: Table S6). These results suggest that histone methylation repatterning by a given marker, although targeted to small proportions and different triad genes, they largely involved genes with common biological functions. We also inspected the chromosomal distribution of the triad genes belonging to different groups in each marker, and we did not observe group-specific or biased distribution (Additional file 1: Figure S5b, S6b).

### Changes of histone methylation in the A and B subgenomes at the hexaploid level following extraction and re-synthesis

The foregoing results indicated that histone modification by both methylation markers to the A and B subgenomes has undergone further changes at the hexaploid level, as being reflected by the clear differences between ETW and both natural wild and domesticated tetraploid wheats (TD265 and TTR13). This raised the question as to whether and to which extent the methylation patterns of the A and B subgenomes in ETW were the same as those in the original hexaploid wheat donor (TAA10) and/or in the resynthesized hexaploid wheat (XX329). To track if histone methylation was altered in the A and B subgenomes following extraction to tetraploid level (in ETW) and then re-synthesis to hexaploid level, we analyzed H3K4me3 and H3K27me3 methylation patterns of the A and B subgenomes for each triad gene in three wheat lines: TAA10 (the hexaploid wheat donor to ETW), ETW, and XX329 (a resynthesized hexaploid wheat with ETW as the maternal parent and *Ae. tauschii* (genome DD) as the paternal parent) (Fig. 1) [26].

**Fig. 5:**
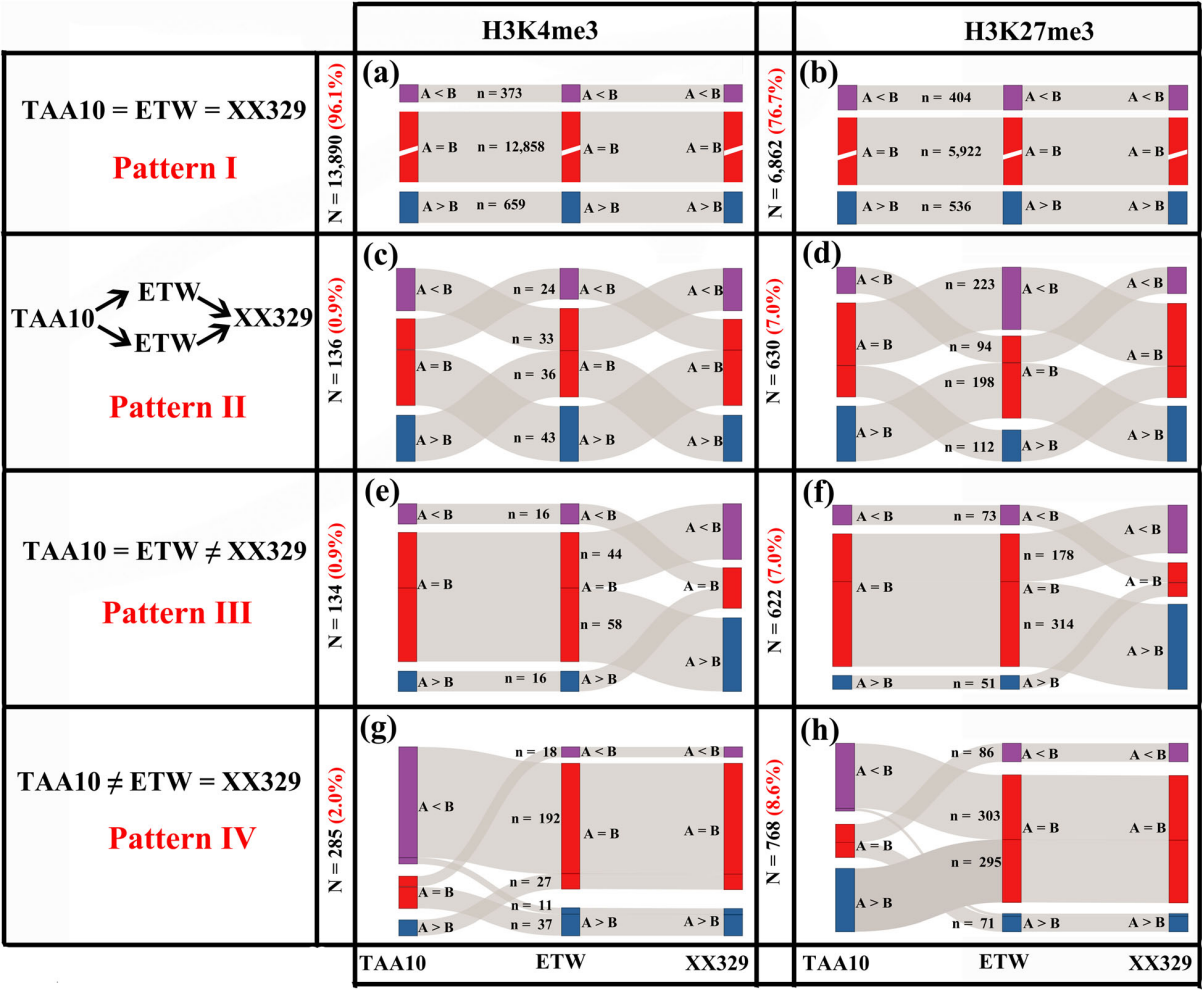
Histone modification patterns between the subgenomes that occurred in the TAA10 → ETW → XX329 ploidy transition process. Histone modification patterns between the A and B subgenomes of the A = B, A > B and A < B patterns in the original hexaploid (TAA10), extracted tetraploid (ETW) and resynthesized hexaploid wheat (XX329). The left panels (**a**, **c**, **e** and **g**) and right panels (**b**, **d**, **f** and **h**) are the H3K4me3 and H3K27me3 markers, respectively. n denotes triad gene number

For this analysis, we categorized the triad genes of the subgenomes A and B homeologs according to their methylation modification by each marker into four patterns, I-IV (Additional file 8: Table S7). Specifically, pattern I (conserved) refers to triad genes whose histone methylation states of each marker remained statistically the same in the three wheat lines, i.e., AABB of TAA10 = ETW = AABB of XX329, indicating their methylation states were not affected during the extraction and reysnthesis processes nor by presence or absence of the D subgenome (Additional file 8: Table S7; Fig. 5a, b). Pattern II (D-genome dependent) refers to triad genes whose histone methylation states changed after dislodging the D subgenome, but which were reverted back upon re-introducing the D genome of *Ae. tauschii* into ETW to form XX329, i.e., AABB of TAA10 ≠ ETW; AABB of TAA10 = AABB of XX329, indicating their methylation states were dependent on presence of the D subgenome (Additional file 8: Table S7; Fig. 5c, d). Pattern III refers to triad genes whose methylation states of each histone marker were unchanged after dislodging the D subgenome, but altered upon the acute reintroduction of the D genome of *Ae. tauschii*, suggesting the changes were due to *trans*-subgenome regulation likely mediated by epigenetic shock (sensu genome shock, McClintock, 1984) [30] when independently evolved distinct genomes are abruptly brought together into a common nucleus/cytoplasm by hybridization. Pattern IV refers to triad genes whose methylation states in each histone marker changed after dislodging the D subgenome, but the patterns remained unchanged after the re-introduction of the D subgenome (i.e., AABB of TAA10 ≠ ETW; ETW = AABB of XX329; AABB of TAA10 ≠ AABB of XX329), suggesting the methylation states of these triad genes in the A and B subgenomes were dependent on the co-evolved D subgenome but not on the acutely reintroduced D subgenome of *Ae. tauschii*, which had never coexisted with the A and B subgenomes. We displayed a subset of representative modification-enriched genomic regions for each of the histone markers (H3K4me3 and H3K27me3) which represented all possible modification patterns as integrative genomics viewer (IGV) snapshots during the TAA10 → ETW → XX329 ploidy transition processes (Fig. 4b; Additional file 1: Figure S4b).

Pattern I (conserved) was by far the most common pattern for both markers. For H3K4me3, 96.1% of triads were conserved, indicating the modification states of this marker in the A and B subgenomes of hexaploid wheat were highly stable and determined almost exclusively *in cis*, that is, uninfluenced by the D subgenome. In contrast, only 76.7% of H3K27me3 markers were conserved, suggesting this marker was less stable than H3K4me3 and influenced to a greater extent by presence of the D subgenome (Additional file 8: Table S7; Fig. 5a, b). Of the remaining three patterns, II and III occupied relatively smaller proportions for both markers, while IV occupied relatively larger proportions for both markers, suggesting intrinsic differences between the co-evolved D subgenome and reintroduced D subgenome of *Ae. tauschii* (Additional file 8: Table S7; Fig. 5c–h). Together, these results indicate that although the A vs. B subgenome histone methylation patterns of the two markers in the vast majority of the triad genes were conserved through the extraction/tetraploidy and re-synthesis/allohexaploidization processes, modifications of certain triad genes were affected by the presence or absence of the D subgenome.

A pattern-based GO analysis of the triad genes indicated that they all showed enriched functional terms. For example, triad genes of H3K4me3-categorized patterns II, III, and IV were involved in alternative oxidase enzyme activities, transporter activity, and different metabolic processes, as well as biological processes such as G protein kinases and various hydrolases (Additional file 1: Figure S7a; Additional file 11: Table S8), while triad genes of H3K27me3-categorized patterns II, III and IV were enriched for mitochondrial inner membrane space, copper ion transport, and copper ion chaperone activity, as well as for MAP kinase activity, CTP synthase activity, ribosome synthesis, and photosynthesis of electronic respiratory chain complexes (Additional file 1: Figure S8a; Additional file 10: Table S9). These results suggest that alterations in histone methylation of the A and B subgenomes at the hexaploid level might be associated with altered molecular activity and cellular metabolism in hexaploid wheat relative to tetraploid wheat. Next, we also analyzed the chromosomal distribution of these triad genes, and we found that while the triad genes for patterns II and III for both markers were evenly distributed across all chromosome arms, genes of pattern IV for both markers were enriched at the distal end of the long arm of chr1 (Additional file 1: Figure S7b, S8b).

### Histone modification levels are dynamic in ploidy transition albeit conservation of patterns

**Fig. 6:**
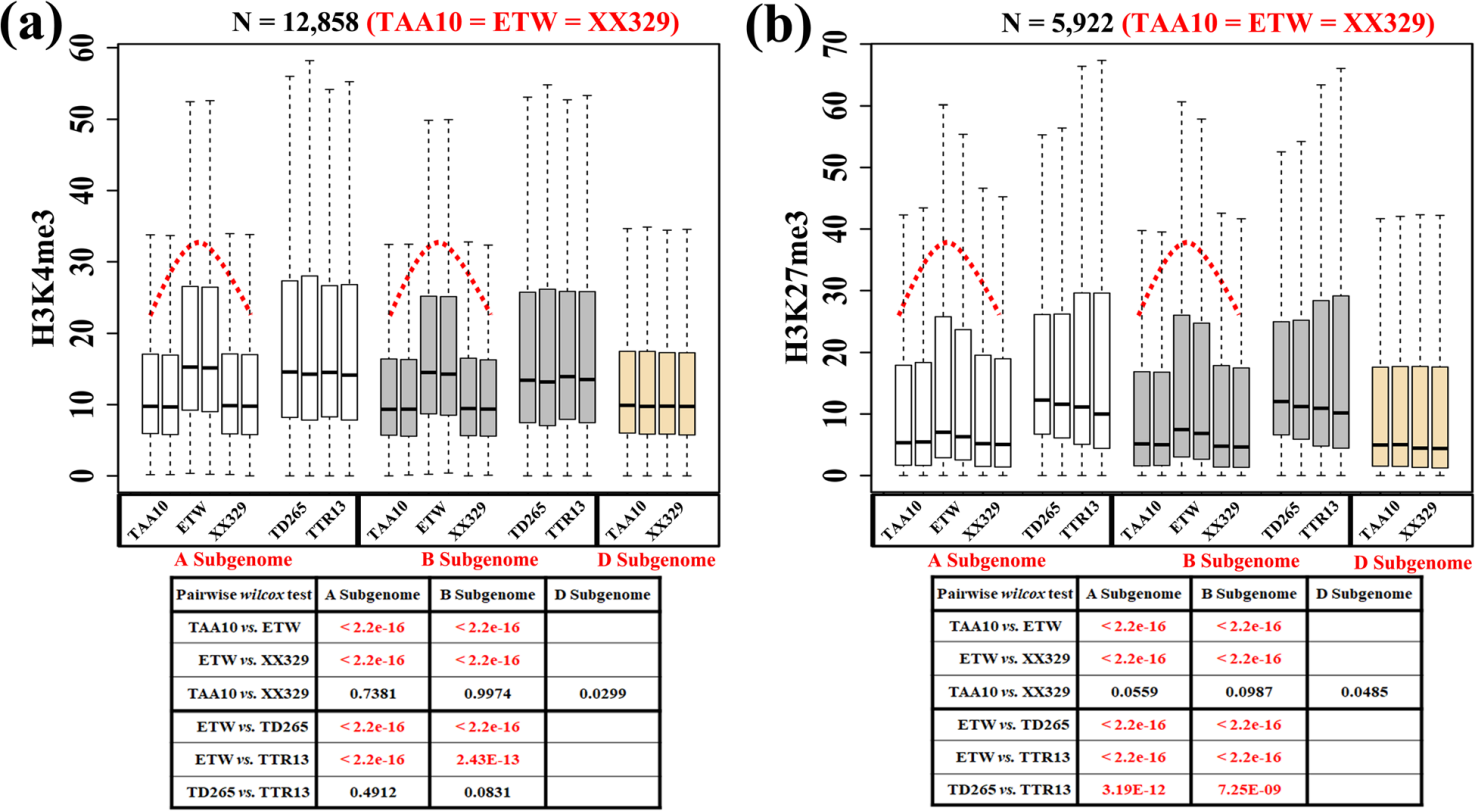
Alteration of histone modification level in the tetraploid extraction and hexaploid re-synthesis process, i.e., TAA10 → ETW → XX329. Only the predominant type (involving majority of the triad gene numbers), i.e., subgenomes A = B in all three genotypes, TAA10 (donor to ETW), ETW (extracted tetraploid wheat) and XX329 (resynthesized hexaploid wheat), for each of the two markers, H3K4me3 (**a**) and H3K27me3 (**b**), was shown, while the rest types were given in Additional file: Supplementary Table S10. The white, gray and yellow-colored boxes indicate expression levels of A-triad, B-triad and D-triad genes, respectively. Significant differences in the *modification level* of both markers is apparent between hexaploid (lower) and tetraploid (higher) wheats, and the alteration was reversible as evidenced by the extraction and reysnthesis process. The Wilcoxon test statistical analysis results are shown for pairwise compassions of subgenomes **a**, **b**, and **d**, below the figures. Red font indicates statistical significance (*p* values < 0.001). The two adjacent boxes are two biological replicates for each genotype

The above analyses identified four patterns of homeologous modification (the modification states of A vs. B homologs for each of the triad genes, i.e., patterns I, II, III, and IV) by the two histone markers. Next, we assessed the overall levels (the enrichment or quantitative level) of histone modifications of the triads identified during the TAA10 → ETW → XX329 ploidy transition processes. We found the overall histone modification levels for both H3K4me3 and H3K27me3 of the triads changed markedly in all four patterns (Additional file 11: Table S10). A major trend of overall modification level changes is a marked increase in ETW relative to the two hexaploid wheat lines, TAA10, the donor to ETW and XX329, the resynthesized hexaploid (Additional file 11: Table S10). This changing trend holds for triad genes of all patterns, which came as a surprise especially for Pattern 1 as in which the modification patterns between the A and B subgenomes were conserved in all comparisons (Fig. 6a, b; Wilcoxon test, *p* value < 0.05). In contrast, there was no difference in overall modification levels between the two hexaploid wheat lines (Wilcoxon test, *p* value > 0.05), indicating changes in the overall modification levels in ETW are due exclusively to *trans*-effects by the D subgenome, which commonly regulate both A and B subgenomes in hexaploid wheat.

We also compared the histone modification levels of ETW to those of natural tetraploid wheat, WTW (TD265) and DTW (TTR13); we found the modification levels for both markers in ETW were significantly higher than both “normal” tetraploid wheat (Wilcoxon test, *p* value < 0.05). There was no difference between TD265 and TTR13 in H3K4me3 histone modification levels (Wilcoxon test, *p* value > 0.05), although they differed in H3K27me3 modification levels (Wilcoxon test, *p* value < 0.05) (Fig. 6a, b). ETW exhibited severe abnormality in all assessed morphological phenotypes [26]; we speculate that this dramatic deterioration in growth and development of ETW might be caused at least in part by the elevated histone modification levels.

### H3K4me3, but not H3K27me3, modifications are significantly correlated with subgenome expression

**Fig. 7:**
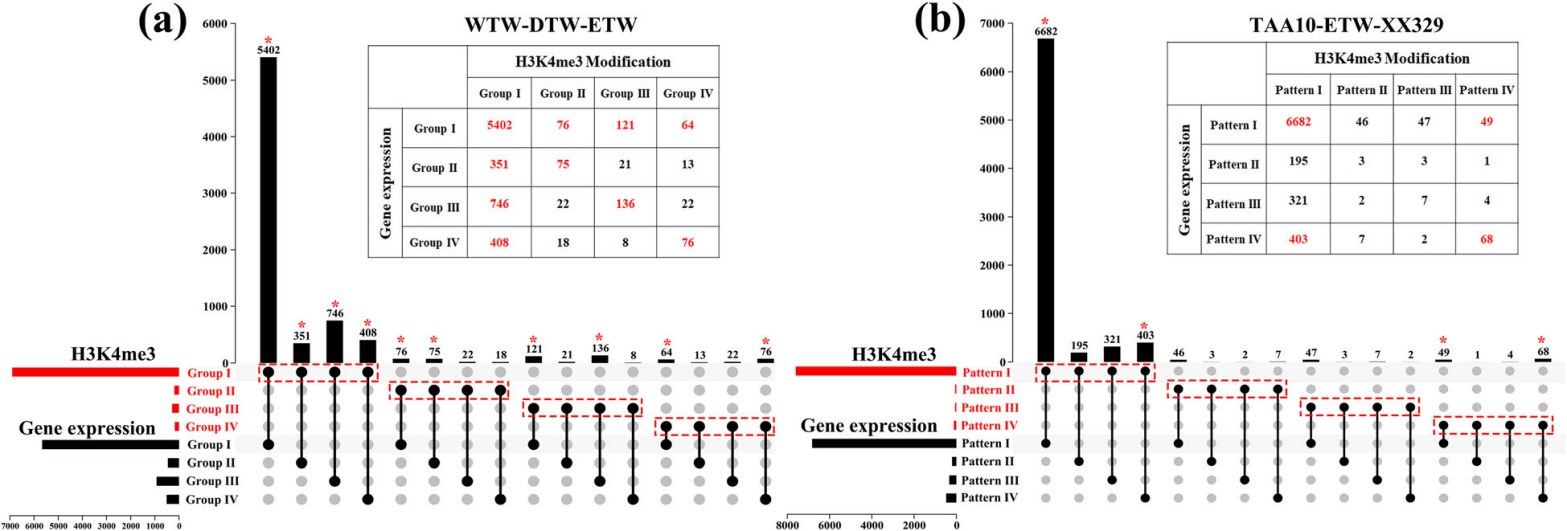
Gene expression is positively correlated with H3K4me3 but not with H3K27me3. The expressed genes were classified into the same categories as those for histone modification analyses, the detailed categories were also shown on different colored lines in different groups. The black dots and the black vertical bars indicate the detected triad patterns, with the total triad gene numbers at the top of the vertical bars. Horizontal bars on the left denote the numbers in each situation, (**a**) and (**b**) refer to H3K4me3 in the WTW → DTW → ETW process and H3K4me3 in the TAA10 → ETW → XX329 process, respectively. An asterisk denotes statistical significance (prop.test, *p* value < 0.05)

The preceding analyses revealed patterns of homeologous modification (the modification states of A vs. B homologs of each of the triad genes). Next, we assessed the relationship between homeologous modification and subgenome expression during the WTW → DTW → ETW process and the TAA10 → ETW → XX329 ploidy transition process. First, we performed similar analyses for expression of the triad genes based on the RNA-seq data of the three tetraploid wheat lines. We found 1719 (19.5%), 2085 (23.7%), and 1927 (21.9%) of the triad genes showed biased expression between the A and B subgenomes in WTW (TD265), DTW (TTR13), and ETW, respectively, and in all three lines subgenome A showed significantly higher expression than B (*p* values < 0.001, Binom test, Additional file 3: Table S2). This indicates that in tetraploid wheat subgenome expression dominance accords with subgenome histone modification dominance. To explore which one or both of the histone markers are underlying this concordance, we checked for overlap and distribution of all the expression-categorized triad gene groups vs. the histone modification-categorized triad gene groups. We found that only H3K4me3 modification showed significant correlations with corresponding triad gene expression (prop.test, *p* values < 0.05) (Fig. 7a), while H3K27me3 did not show significant correlation in any of the gene groups (*p* values > 0.05) (Additional file 1: Figure S9a). Similar results were also found during the extraction and re-synthesis process (Fig. 7b, Additional file 1: Figure S9b).

## Discussion

Changes in transcriptional gene expression and genome regulatory framework accompanying allopolyploidization have been studied in many allopolyploid systems, from the model plant Arabidopsis [31–33] to wheat [17, 29, 34–38], cotton [20, 24, 39, 40], rape [21, 41–44], *Senecio* [45], *Spartina* [46], and *Tragopogon* [47, 48]. The unraveled changes encompass a plethora of phenomena, including homoeolog expression bias, subgenome dominance, expression-level dominance, and remodeling of co-expression networks [9, 13, 14, 49–51]. Notwithstanding the impressive progress made in this research area, how histone modifications affect and modify subgenome(s) in isolation, and how these modifications affect gene expression during evolutionary processes, remain largely unexplored for most species. Here, we employed a set of tetraploid and hexaploid wheat materials with known evolutionary and genetic relationships as a model to investigate the stability and dynamics of two typical euchromatin histone modifications (H3K4me3 and H3K27me3) in relation to polyploid formation, evolution, and selection under domestication, and their attendant impacts on subgenome gene expression.

We found that the A-subgenome in all three tetraploid wheats showed higher levels of modifications for both histone modification markers except in ETW by H3K27me3. Although the underlying mechanism of this generic conservation remains unclear, it is possible that this phenomenon (asymmetric histone modifications in A vs. B methylation patterns) is an important intermediate transitional state during the process of allopolyploid evolution. Epigenetic effects are not only common, but can also underlie or contribute to many aspects of plant evolution because its effects can be persistent across many generations, i.e., transgenerational epigenetic inheritance [52, 53]. Our finding of A-subgenome modification dominance in tetraploid wheat by the histone markers is consistent with previous results on gene expression, which showed the A-subgenome displaying an overall higher expression than B-subgenome in both wild and domesticated tetraploid wheat in both leaf and floret tissues [29], suggesting asymmetry between subgenomes in terms of both epigenetic modifications and gene expression may play a significant role in neo-allopolyploids, i.e., before the onset of large-scale diploidization [38, 39, 47]. However, there are still many unanswered questions, including the mechanism by which subgenome asymmetry arises and maintains, and how polyploids “decide” which subgenome will be dominant given the high sequence similarity between the homoeologous subgenomes in general and the syntenic homoeologous gene triads in particular.

Harboring the AABB component of bread wheat, the extracted tetraploid wheat (ETW) was previously found to have extensive transcriptomic alterations compared to the wild and domesticated tetraploid wheats as a result of its residence at the allohexaploid level over evolutionary time, and which in turn caused phenotypic abnormalities [26]. Here, we found that histone modifications by the two markers also exhibited extensive and, to an extent, distinct changes in the AABB component of bread wheat. We also distinguished the tetraploid level effects from that of the hexaploid level during the evolutionary journey, and dissected the status of all the histone modifications. We found that 70.9–88.3% of triad genes can maintain their histone modification patterns in the process of domestication and evolution under cultivation at both tetraploid and hexaploid levels. This broadly accords with a recent study that analyzed profiles of two histone methylation markers, H3K27me2 and H3K27me3, in natural tetraploid and hexaploid wheats, and found that while H3K27me2 was affected by ploidy levels, H3K27me3 was largely not [54]. However, slightly different from observations by this study [54], we found small proportions of triad genes bearing modifications by both H3K4me3 and H3K27me3 markers to the A and B subgenomes have undergone further changes at the hexaploid level, but H3K4me3 is more stable than H3K27me3. This was reflected by clear differences between ETW (harboring the AABB subgenomes of hexaploid common wheat), and wild tetraploid wheat, *T. turgidum* ssp. *dicoccoides* (TD265) and domesticated tetraploid wheat, ssp. *durum* (TTR13). Gene Ontology (GO) analyses indicated that triad genes that bear hexaploidy-incurred further modification changes are enriched in diverse biological pathways and processes, including cellular signaling, response to desiccation, catalytic activities. Based on these results, we suspect that subgenome asymmetry has been markedly reinforced over long-term evolutionary and domestication/breeding processes. The chromatin and epigenetic modifications identified between subgenomes in the three types of tetraploid wheats may provide new insights into the interpretation of gene expression asymmetry between different subgenomes.

The aforementioned results raise the question as to whether and to which extent the histone methylation patterns of the A and B subgenomes in ETW were the same as those in the original hexaploid wheat (TAA10) that donated the ETW and in the resynthesized hexaploid. In the ploidy-transition processes, i.e., from TAA10 (hexaploid) to ETW (tetraploid) and back to XX329 (hexaploid), we found that 76.7% - 96.1% of triad genes maintained stable levels across the two histone modifications, indicating they were regulated by *cis*-acting modules and were therefore predominantly autonomous in the AABB genomes. The remaining 3.9% - 23.3% of triad genes showed variable levels in the ploidy-transition processes, suggesting their dependency on *trans*-regulatory effects contributed by the D subgenome in hexaploid wheat. Genome autonomy has rarely been investigated, with the notable exception of studies in Arabidopsis [33], cotton [40], and wheat [37], which have indicated that *trans*-regulation within polyploid genomes results from a complex interplay between epigenetic modification and gene expression regulation at multiple levels. As the dynamic nature of the D progenitor genome accompanies speciation by interspecific hybridization, the extraction of the AABB subgenomes from a hexaploid wheat provides a unique opportunity to study genome evolution and interplay between subgenomes. Our findings reveal an unambiguous and well-classified basis for regulation in the interplay between the AABB subgenomes and the DD genome. This effect included at least three epigenetic regulation patterns: one involves exclusive dependency on the DD subgenome (can be completely reversible), and the other two are the prompt *trans*-subgenome regulation mediated by the presence of a novel DD genome and the distinct evolutionary functions mediated by co-existence with the DD genome, indicating that the interaction between these subgenomes not only affects AABB subgenomes, but also the DD genome. One intriguing phenomenon is that genes in Pattern IV show aggregation at the distal end of the long arm of chr1, which was enriched in mitochondrial respiratory chain pathway genes and various kinase and complex activities; these results may partially explain why ETW showed a series of abnormal phenotypes with dwarfed stature, decreased number of tillers and reduced seed production [26]. Of note, the histone modification levels of H3K4me3 and H3K27me3 were increased in ETW and the increased histone modification levels were restored to the original level (TAA10) in resynthesized XX329, which suggests that not only the histone modification patterns but also the histone modification levels have changed during the ploidy transition processes. Using the same set of plant lines, a recent study showed that DNA methylation was also substantially decreased in ETW but reverted back in the resynthesized XX329 [55]. Collectively, these findings all point to the multi-level regulatory architecture brought about by epigenetic mechanisms accompanying allopolyploid speciation and evolution.

Finally, as expected, H3K4me3 modification was positively correlated with subgenomic gene expression, while H3K27me3 modification was not significantly correlated with subgenomic gene expression in either the domestication (WTW → DTW → ETW) or the ploidy transition (TAA10 → ETW → XX329) processes [56–60]. It has been well-established in plants, as in animals, that H3K27me3 plays crucial roles during major developmental phase transitions by targeted repressing spatiotemporal expression of developmental genes dictating the standing tissue phase but not the derivative one (reviewed in [61]). Thus, one explanation for the lack of a correlation between H3K27me3 modification and subgenome gene expression we observed in this study is likely because we analyzed only one tissue (leaf), which is a fully differentiated tissue and does not invoke developmental phase transitions. That the enriched GO terms of the H3K27me3-differentially modified triad genes are mainly involved in basic nuclear activities (e.g., DNA and protein synthesis), ribosome synthesis, cellular metabolism, growth and photosynthesis, corroborates this possibility. Likewise, this explanation also helps to rule out the concern that some of the differentially modified triad genes by the two histone markers are due to secondary effects of developmental differences across the wheat lines rather than the direct result of the evolution/domestication or the ploidy level transition process.

## Conclusion

We explored patterns and levels of histone methylation in the A and B subgenomes during the evolution and domestication of tetraploid and hexaploid wheat, as well as during the hexaploidy-tetraploidy-hexaploidy ploidy level transition process. This was done by contrasting the regulatory variations between A and B syntenic homoeologous triad genes. Our observations lead to the conclusion that an increased opportunity for *trans*-regulation accompanying the introduction of additional subgenomes (e.g., from diploid to allotetraploid to allohexaploid) might be a general and previously under-appreciated feature of evolution via allopolyploidy, which contributes to evolutionary novelty in neo-allopolyploids, and bears implications for the genetic improvement and de novo domestication of allopolyploid crops especially evoking the “synthetic” approaches.

## Methods

### Plant materials

The materials used in this study were a wild tetraploid wheat (*T. turgidum*, ssp. *dicoccoides*, line TD265), a domesticated tetraploid wheat (*T. turgidum*, ssp. *durum*, cv. TTR13), an extracted tetraploid wheat (ETW) representing the AABB component of common wheat, a resynthesized hexaploid wheat designated XX329 (produced by crossing ETW with *Ae. tauschii*, line TQ18), and a common wheat cultivar TAA10 from which the ETW was extracted [26, 27]. The relationship between the six plant lines was shown in Fig. 1. All materials in the experiment were grown in an artificial climate chamber under the conditions of 24/20 °C day/night and 16 h day length. The leaf was sampled from four-leaf stage seedlings, frozen immediately in liquid nitrogen, and stored in − 80 °C freezer until use.

### ChIP-seq library preparation and data analysis

Chromatin immunoprecipitation (ChIP) was performed according to an established protocol with slight modifications [62]. For each genotype, nuclei were isolated from 2 g of the leaf tissue, and digested with micrococcal nuclease (Sigma, cat. # N3755). For each genotype, we employed two biological replicates for each histone modification markers, H3K4me3 (Millipore, cat. # 07-473) and H3K27me3 (Millipore, cat. # 07-449) antibodies. The 150 bp pair-end ChIP-seq reads were obtained and the detailed information was listed in Additional file 2: Table S1. The sequences generated by ChIP-seq were aligned to the wheat reference genome (CS, RefSeq v1.0) using the hisat2 software using default parameters [63, 64]. To ensure accuracy and reliability of our analyses, we only extracted the unique reads (only a single matching site on the reference genome) for subsequent analyses. There are 110,790 annotated HC (high confidence) genes in the CS reference genome, but we only used the triad gene dataset (17,753 pairs of triads) as a basis for our analysis. The BAM files generated by the sequence alignment were used to obtain the original reads on each gene by using the bedtools software [65] and then converted to the corresponding TPM (Transcripts Per Kilobase Million) value by the TBtools software [66].

### RNA-sequencing and data analyses

Total RNAs were extracted using Trizol reagent (Invitrogen) and sequenced by the HiSeq2500 platform. The 150 bp pair-end RNA-seq libraries with average insert size of 300 bp were constructed and then sequenced on Illumina HiSeq2500 platform, and clean reads obtained (Table S1). Clean reads were then mapped to the reference genome (RefSeq v1.0, [27]) using hisat2 software with default parameters [28, 63]. Only unique mapped reads were used for obtaining the original reads and TBtools software was used to convert into the corresponding TPM value [66]. We used a similar processing method as for the ChIP-seq to assess triad expression patterns in RNA-Seq data.

### Gene ontology (GO) analysis

GO term enrichment test was performed using the GoSlim annotation file generated by our lab, in which GO terms with FDR-controlled *q* value < 0.05 was defined as a significantly enriched GO category.

### Statistical analysis

The statistical significance of each comparison and graphical analysis were executed in R (version 3.5.1). The correlations between two biological replicates and among the five plant lines were assessed using the Pearson correlation coefficient based on TPM in R. We found the correlations between the two biological replicates had average coefficients of 0.99 and 0.91 for H3K4me3 and H3K27me3, respectively (Additional file 1: Figure S2).

To quantify relative proportions of the three patterns for each marker between the A and B subgenomes (A = B, A > B or A < B) in each of the three tetraploid wheat lines, the R package “rdist” was used to analyze the statistical significance of the histone modification patterns between the A, and B subgenomic triads according to previously published methods [27]: (A = B) = c (0.5, 0.5); (A > B) = c (1, 0); A < B = c (0, 1), that is, the Euclidean distance using the Pythagorean theorem or the Pythagorean distance (Additional file 3: Table S2). After defining the three patterns (A = B, A > B or A < B), we tracked the different patterns from wild tetraploid wheat (WTW, accession TD265) to domesticated tetraploid wheat (DTW, cv. TTR13), and then to the extracted tetraploid wheat (ETW), and categorized all triad genes into four groups (Fig. 3; Additional file 5: Table S4). Similar analyses were performed for the TAA10 → ETW → XX329 ploidy transition processes. For the two hexaploid wheat lines, we focused only on the A and B subgenomes. Finally, we classified all the analyzed triad genes into four patterns (Fig. 5; Additional file 8: Table S7).

To assess the overall levels of histone modifications of the triads identified during the TAA10 → ETW → XX329 ploidy transition process, a Wilcoxon test was applied with a *p* value of 0.05 as a cutoff for significance. We found the overall histone modification levels for both H3K4me3 and H3K27me3 of the triads changed markedly across all four patterns (detailed information in Fig. 6; Additional file 11: Table S10).

To determine whether the two histone modifications were correlated with gene expression, a prop.test was applied separately for each Group/Pattern for the WTW → DTW → ETW process and for the TAA10 → ETW → XX329 ploidy transition process with a 0.05 *p* value as a cutoff (Fig. 7; Additional file 1: Figure S9).

## Supplementary Information

**Additional file 1: Figure S1.** The relative proportions of raw data mapped to the three subgenomes of hexaploid wheat. (**a**), (**b**) and (**c**) are the H3K4me3 ChIP-seq data, H3K27me3 ChIP-seq data and RNA-seq data, respectively. **Figure S2.** Visualized correlation coefficients between the two biological replicates of the ChIP-seq data for the two histone markers and peaks of H3K4me3 and H3K27me3 at representative triad genes. (**a**) AABB components in H3K4me3; (**b**) Subgenome A in H3K4me3; (**c**) Subgenome B in H3K4me3; (**d**) AABB components in H3K27me3; (**e**) Subgenome A in H3K27me3; (**f**) Subgenome B in H3K27me3; (**g**) peaks of H3K4me3 in subgenomes A, B and D (from left to right in each panel); (**h**) peaks of H3K27me3 in subgenomes A, B and D (from left to right in each panel). **Figure S3.** Genome and chromosomal distribution of H3K27me3 in all the plant lines used in this study. Denotations are the same as in legend to Fig. 2. **Figure S4.** Examples of conservation and remodeling of the two histone modifications (H3K4me3 and H3K27me3) shown by integrative genomics viewer (IGV) snapshots. (**a**) Groups I-IV H3K4me3 histone modification patterns during the WTW → DTW → ETW process. For Group I (conserved), the three possible relationships, namely, A = B, A < B and A > B, in each of the WTW → DTW → ETW steps are presented; Group II shows changes that occurred in DTW and maintained in ETW; Group III shows changes that occurred in DTW and changed further in ETW; Group IV shows changes that only occurred in ETW. (**b**) Groups I-IV histone modification patterns (H3K27me3) in the TAA10 → ETW → XX329 ploidy transition process. For Pattern I (conserved), all three changing patterns, namely, A = B, A < B and A > B, in each of the TAA10 → ETW → XX329 steps are presented; Pattern II indicates reversible changes that are exclusively dependent on the presence of DD subgenome; Pattern III indicates changes that only occurred in XX329, reflecting prompt *trans*-subgenome regulation mediated by the presence of a novel DD genome (newly introduced from *Aegilops tauchii*); Pattern IV indicates changes that occurred in ETW and XX329, suggesting possible distinct functions of the AABB subgenome. **Figure S5.** Gene Ontology (GO) analyses of group II-IV genes bearing H3K4me3 modification in WTW → DTW → ETW process (**a**), and their genome and chromosome distributions (**b**). From the outside to the inside in (**b**): Group IV, Group III, Group II, and Group I gene distributions: the blue color represents all genes distributed along the chromosomes. The outermost layer is the position information of the chromosome, where yellow denotes the centromeric and pericentromeric region (Proximal (C)), light blue represents the chromosome arm (Interstitial (R2a/R2b)), and the dark blue indicates the end of the chromosome (Distal (R1/R3)). The punctuated innermost red lines represent length of all the seven wheat chromosomes [28]. **Figure S6.** Gene Ontology (GO) analyses of group II-IV genes of H3K27me3 modifications in the WTW → DTW → ETW process (**a**) and gene distribution on genome (**b**). From the outside to the inside in (**b**) are distribution of the genes categorized to Groups IV, III, II, and I, respectively. Blue color denotes the distribution of all genes along the chromosomes. The outermost layer represents the position information of the chromosome, where pale yellow shows the centromeric and pericentromeric regions (Proximal (C)), light blue represents chromosome arm (Interstitial (R2a/R2b)), and dark blue indicates chromosome end (Distal (R1/R3)). The punctuated innermost red lines represent length of all the seven wheat chromosomes [28]. **Figure S7.** Gene Ontology (GO) analyses of pattern II-IV genes of H3K4me3 modification in TAA10 → ETW → XX329 process and gene distribution on genome. From the outside to the inside: Pattern IV, Pattern III, Pattern II, and Pattern I gene distributions, then the blue color represents the distribution of all genes along the chromosomes. The outermost layer is the position information of the chromosome, where the pale yellow shows the centromere and the pericentromeric region (Proximal (C)), the light blue represents the chromosome arm (Interstitial (R2a/R2b)), and the dark blue indicates the end of the chromosome (Distal (R1/R3)). The innermost red line represents the length of all the chromosomes. Black arrows indicate genes of pattern IV, which were enriched at the distal end of the long arm of chr1A and chr1B. **Figure S8.** Gene Ontology (GO) analyses of pattern II-IV genes of H3K27me3 modification in TAA10 → ETW → XX329 process and gene distribution on genome. From the outside to the inside: Pattern IV, Pattern III, Pattern II, and Pattern I genes distribution, then the blue color represents the distribution of all genes along the chromosome. The outermost layer is the position information of the chromosome, where the pale yellow shows the centromere and the pericentromeric region (Proximal (C)), the light blue represents the chromosome arm (Interstitial (R2a/R2b)), and the dark blue indicates the end of the chromosome (Distal (R1/R3)). The innermost red line represents the length of all the chromosomes. Black arrows indicate genes of pattern IV, which were enriched at the distal end of the long arm of chr1A and chr1B. **Figure S9.** Gene transcription is not positively correlated H3K27me3 in a comparison of A and B subgenomes between WTW → DTW → ETW process and also TAA10 → ETW → XX329 process.

**Additional file 2: Table S1**. Information related to data mapping for ChIP-seq and RNA-Seq.

**Additional file 3: Table S2**. The number of genes analyzed in wild, domesticated and extracted tetraploid wheat.

**Additional file 4: Table S3**. Pairwise comparison of three modes of histone modification in wild, domesticated and extracted tetraploid wheat.

**Additional file 5: Table S4.** The triad genes analyzed in wild, domesticated and extracted tetraploid wheat.

**Additional file 6: Table S5**. Gene Ontology (GO) enrichment terms and genes used for GO analysis of H3K4me3 modification in WTW → DTW → ETW process.

**Additional file 7: Table S6**. Gene Ontology (GO) enrichment terms and genes used for GO analysis of H3K27me3 modification in WTW → DTW → ETW process.

**Additional file 8: Table S7**. The transformation of genes analyzed in the hexaploidy-extracted tetraploid – resynthesized hexaploidy, TAA10 → ETW → XX329 ploidy transition process.

**Additional file 9: Table S8**. The pairwise *wilcox* test analyzed in the hexaploidy-extracted tetraploid –resynthesized hexaploidy, TAA10 → ETW → XX329 ploidy transition process.

**Additional file 10: Table S9**. Gene Ontology (GO) enrichment terms and genes used for GO analysis of H3K4me3 modification in TAA10 → ETW → XX329 ploidy transition process.

**Additional file 11: Table S10**. Gene Ontology (GO) enrichment terms and genes used for GO analysis of H3K27me3 modification in TAA10 → ETW → XX329 ploidy transition process.

## Data Availability

The ChIP-seq data and RNA-Seq data sets have been submitted to NCBI under the accession number PRJNA52835 or via the website: https://www.ncbi.nlm.nih.gov/sra/PRJNA528351 [67].
