# Supplementary figures and images for: Conservation and trans-regulation of histone modification in the A and B subgenomes of polyploid wheat during domestication and ploidy transition
Source: BMC Biol. 2021 Mar 9;19:42. doi: 10.1186/s12915-021-00985-7 (PMC7944620; doi:10.1186/s12915-021-00985-7)

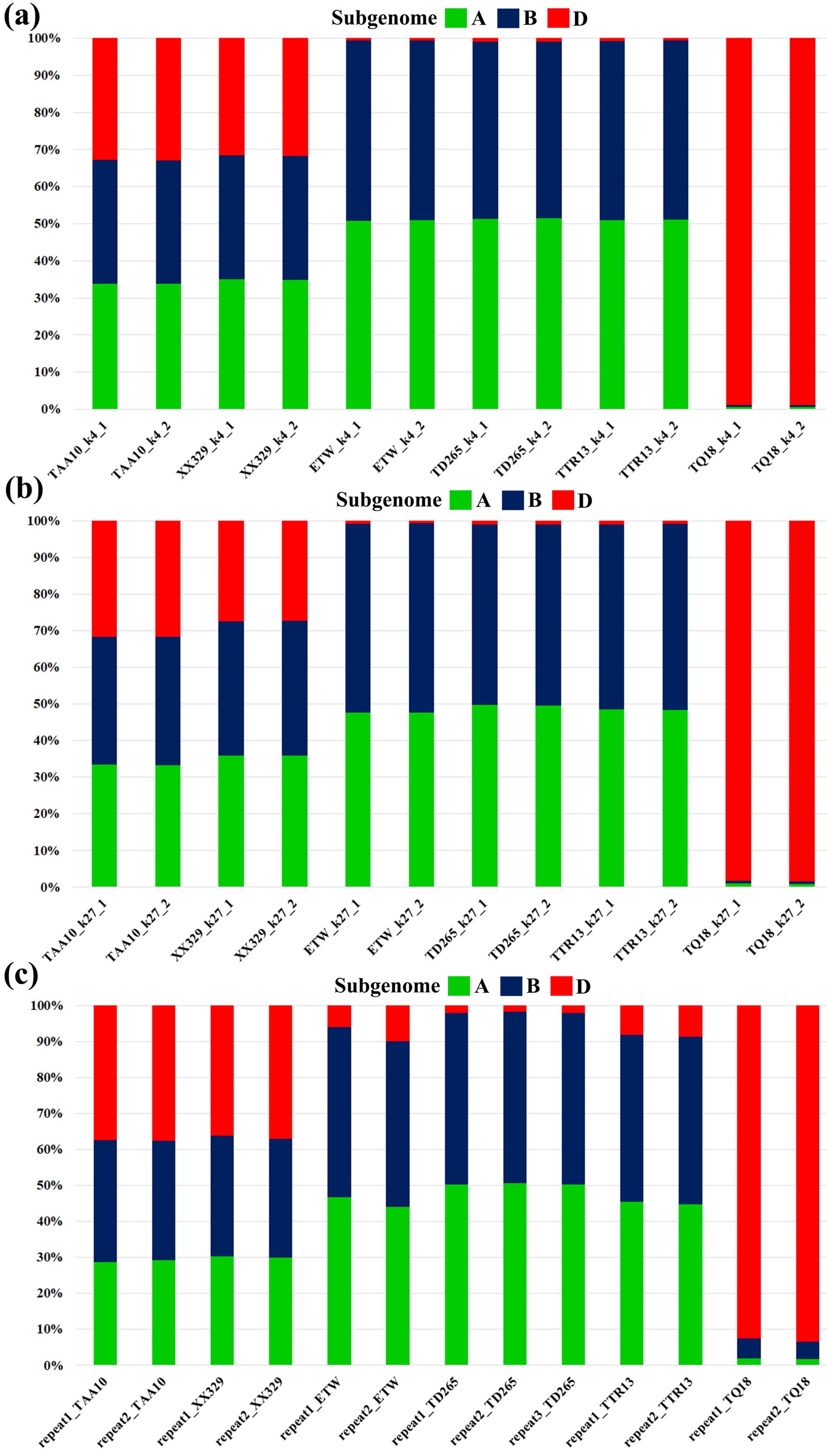

Supplement: Supplementary file 1 — Additional file 1: Figure S1. The relative proportions of raw data mapped to the three subgenomes of hexaploid wheat. (a), (b) and (c) are the H3K4me3 ChIP-seq data, H3K27me3 ChIP-seq data and RNA-seq data, respectively. Figure S2. Visualized correlation coefficients between the two biological replicates of the ChIP-seq data for the two histone markers and peaks of H3K4me3 and H3K27me3 at representative triad genes. (a) AABB components in H3K4me3; (b) Subgenome A in H3K4me3; (c) Subgenome B in H3K4me3; (d) AABB components in H3K27me3; (e) Subgenome A in H3K27me3; (f) Subgenome B in H3K27me3; (g) peaks of H3K4me3 in subgenomes A, B and D (from left to right in each panel); (h) peaks of H3K27me3 in subgenomes A, B and D (from left to right in each panel). Figure S3. Genome and chromosomal distribution of H3K27me3 in all the plant lines used in this study. Denotations are the same as in legend to Fig. 2. Figure S4. Examples of conservation and remodeling of the two histone modifications (H3K4me3 and H3K27me3) shown by integrative genomics viewer (IGV) snapshots. (a) Groups I-IV H3K4me3 histone modification patterns during the WTW → DTW → ETW process. For Group I (conserved), the three possible relationships, namely, A = B, A < B and A > B, in each of the WTW → DTW → ETW steps are presented; Group II shows changes that occurred in DTW and maintained in ETW; Group III shows changes that occurred in DTW and changed further in ETW; Group IV shows changes that only occurred in ETW. (b) Groups I-IV histone modification patterns (H3K27me3) in the TAA10 → ETW → XX329 ploidy transition process. For Pattern I (conserved), all three changing patterns, namely, A = B, A < B and A > B, in each of the TAA10 → ETW → XX329 steps are presented; Pattern II indicates reversible changes that are exclusively dependent on the presence of DD subgenome; Pattern III indicates changes that only occurred in XX329, reflecting prompt trans-subgenome regulation mediated by the presence of a novel DD [file 12915_2021_985_MOESM1_ESM.zip › 12915_2021_985_MOESM1_ESM/Figure S1_ESM.pdf]

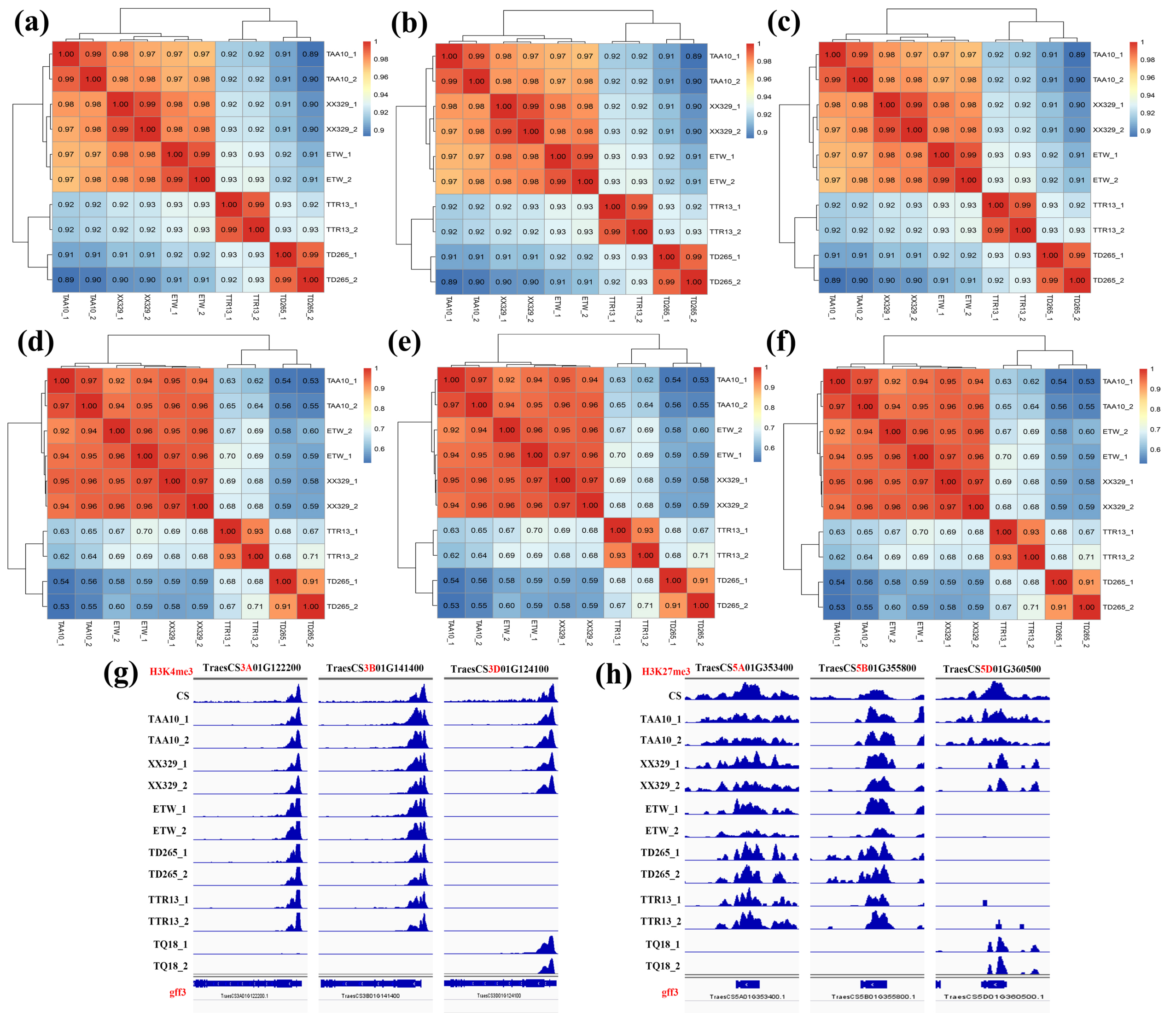

Supplement: Supplementary file 1 — Additional file 1: Figure S1. The relative proportions of raw data mapped to the three subgenomes of hexaploid wheat. (a), (b) and (c) are the H3K4me3 ChIP-seq data, H3K27me3 ChIP-seq data and RNA-seq data, respectively. Figure S2. Visualized correlation coefficients between the two biological replicates of the ChIP-seq data for the two histone markers and peaks of H3K4me3 and H3K27me3 at representative triad genes. (a) AABB components in H3K4me3; (b) Subgenome A in H3K4me3; (c) Subgenome B in H3K4me3; (d) AABB components in H3K27me3; (e) Subgenome A in H3K27me3; (f) Subgenome B in H3K27me3; (g) peaks of H3K4me3 in subgenomes A, B and D (from left to right in each panel); (h) peaks of H3K27me3 in subgenomes A, B and D (from left to right in each panel). Figure S3. Genome and chromosomal distribution of H3K27me3 in all the plant lines used in this study. Denotations are the same as in legend to Fig. 2. Figure S4. Examples of conservation and remodeling of the two histone modifications (H3K4me3 and H3K27me3) shown by integrative genomics viewer (IGV) snapshots. (a) Groups I-IV H3K4me3 histone modification patterns during the WTW → DTW → ETW process. For Group I (conserved), the three possible relationships, namely, A = B, A < B and A > B, in each of the WTW → DTW → ETW steps are presented; Group II shows changes that occurred in DTW and maintained in ETW; Group III shows changes that occurred in DTW and changed further in ETW; Group IV shows changes that only occurred in ETW. (b) Groups I-IV histone modification patterns (H3K27me3) in the TAA10 → ETW → XX329 ploidy transition process. For Pattern I (conserved), all three changing patterns, namely, A = B, A < B and A > B, in each of the TAA10 → ETW → XX329 steps are presented; Pattern II indicates reversible changes that are exclusively dependent on the presence of DD subgenome; Pattern III indicates changes that only occurred in XX329, reflecting prompt trans-subgenome regulation mediated by the presence of a novel DD [file 12915_2021_985_MOESM1_ESM.zip › 12915_2021_985_MOESM1_ESM/Figure S2_ESM.pdf]

# H3K27me3

Input-CS

TQ18-1

TQ18-2

TD265-1

TD265-2

TTR13-1

TTR13-2

ETW-1

ETW-2

XX329-1

XX329-2

TAA10-1

TAA10-2

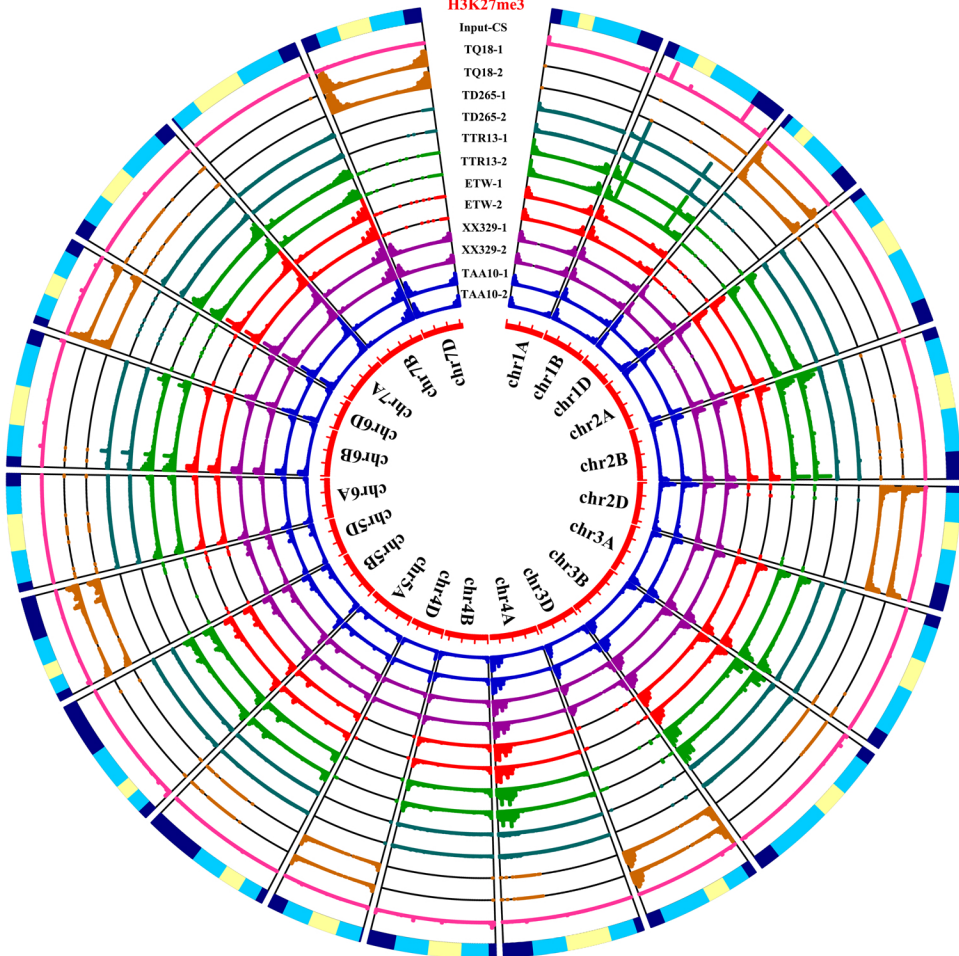

Supplement: Supplementary file 1 — Additional file 1: Figure S1. The relative proportions of raw data mapped to the three subgenomes of hexaploid wheat. (a), (b) and (c) are the H3K4me3 ChIP-seq data, H3K27me3 ChIP-seq data and RNA-seq data, respectively. Figure S2. Visualized correlation coefficients between the two biological replicates of the ChIP-seq data for the two histone markers and peaks of H3K4me3 and H3K27me3 at representative triad genes. (a) AABB components in H3K4me3; (b) Subgenome A in H3K4me3; (c) Subgenome B in H3K4me3; (d) AABB components in H3K27me3; (e) Subgenome A in H3K27me3; (f) Subgenome B in H3K27me3; (g) peaks of H3K4me3 in subgenomes A, B and D (from left to right in each panel); (h) peaks of H3K27me3 in subgenomes A, B and D (from left to right in each panel). Figure S3. Genome and chromosomal distribution of H3K27me3 in all the plant lines used in this study. Denotations are the same as in legend to Fig. 2. Figure S4. Examples of conservation and remodeling of the two histone modifications (H3K4me3 and H3K27me3) shown by integrative genomics viewer (IGV) snapshots. (a) Groups I-IV H3K4me3 histone modification patterns during the WTW → DTW → ETW process. For Group I (conserved), the three possible relationships, namely, A = B, A < B and A > B, in each of the WTW → DTW → ETW steps are presented; Group II shows changes that occurred in DTW and maintained in ETW; Group III shows changes that occurred in DTW and changed further in ETW; Group IV shows changes that only occurred in ETW. (b) Groups I-IV histone modification patterns (H3K27me3) in the TAA10 → ETW → XX329 ploidy transition process. For Pattern I (conserved), all three changing patterns, namely, A = B, A < B and A > B, in each of the TAA10 → ETW → XX329 steps are presented; Pattern II indicates reversible changes that are exclusively dependent on the presence of DD subgenome; Pattern III indicates changes that only occurred in XX329, reflecting prompt trans-subgenome regulation mediated by the presence of a novel DD [file 12915_2021_985_MOESM1_ESM.zip › 12915_2021_985_MOESM1_ESM/Figure S3_ESM.pdf]

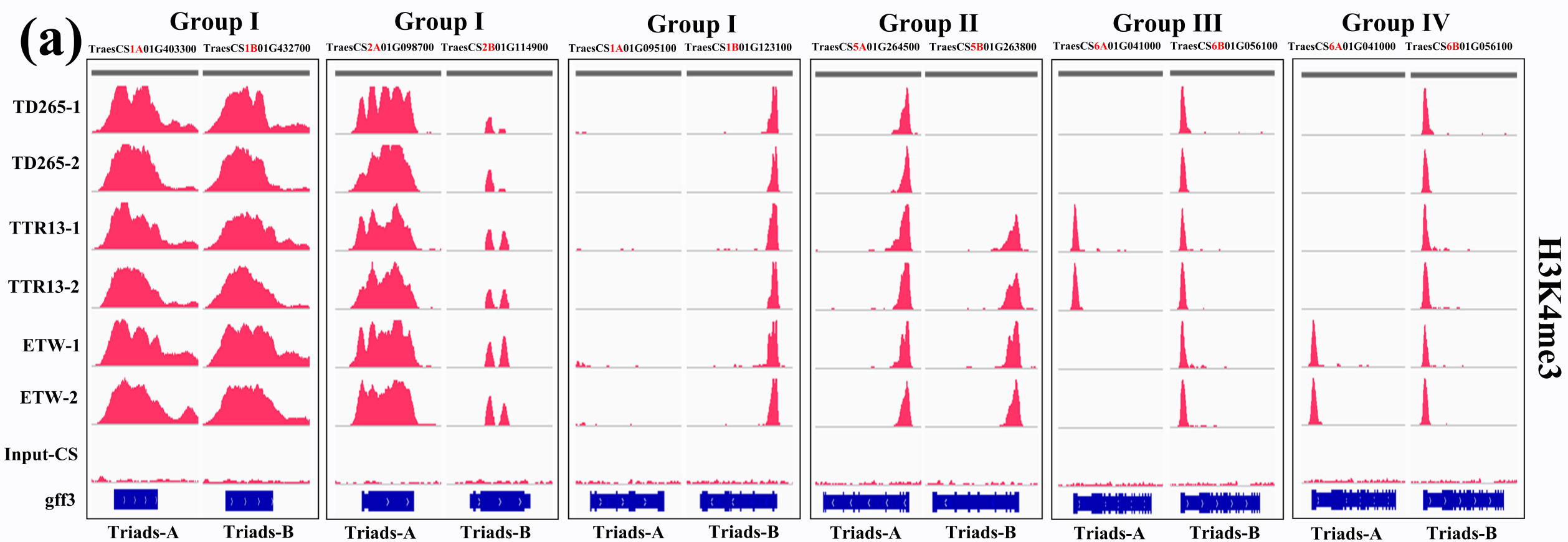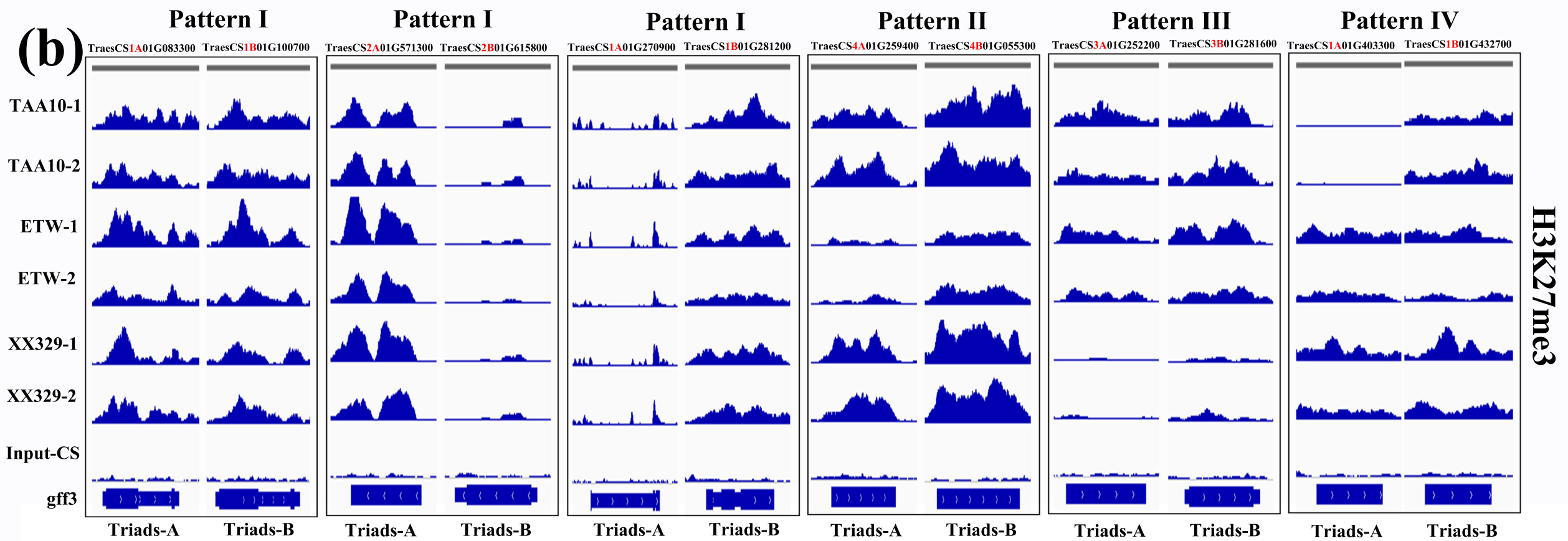

Supplement: Supplementary file 1 — Additional file 1: Figure S1. The relative proportions of raw data mapped to the three subgenomes of hexaploid wheat. (a), (b) and (c) are the H3K4me3 ChIP-seq data, H3K27me3 ChIP-seq data and RNA-seq data, respectively. Figure S2. Visualized correlation coefficients between the two biological replicates of the ChIP-seq data for the two histone markers and peaks of H3K4me3 and H3K27me3 at representative triad genes. (a) AABB components in H3K4me3; (b) Subgenome A in H3K4me3; (c) Subgenome B in H3K4me3; (d) AABB components in H3K27me3; (e) Subgenome A in H3K27me3; (f) Subgenome B in H3K27me3; (g) peaks of H3K4me3 in subgenomes A, B and D (from left to right in each panel); (h) peaks of H3K27me3 in subgenomes A, B and D (from left to right in each panel). Figure S3. Genome and chromosomal distribution of H3K27me3 in all the plant lines used in this study. Denotations are the same as in legend to Fig. 2. Figure S4. Examples of conservation and remodeling of the two histone modifications (H3K4me3 and H3K27me3) shown by integrative genomics viewer (IGV) snapshots. (a) Groups I-IV H3K4me3 histone modification patterns during the WTW → DTW → ETW process. For Group I (conserved), the three possible relationships, namely, A = B, A < B and A > B, in each of the WTW → DTW → ETW steps are presented; Group II shows changes that occurred in DTW and maintained in ETW; Group III shows changes that occurred in DTW and changed further in ETW; Group IV shows changes that only occurred in ETW. (b) Groups I-IV histone modification patterns (H3K27me3) in the TAA10 → ETW → XX329 ploidy transition process. For Pattern I (conserved), all three changing patterns, namely, A = B, A < B and A > B, in each of the TAA10 → ETW → XX329 steps are presented; Pattern II indicates reversible changes that are exclusively dependent on the presence of DD subgenome; Pattern III indicates changes that only occurred in XX329, reflecting prompt trans-subgenome regulation mediated by the presence of a novel DD [file 12915_2021_985_MOESM1_ESM.zip › 12915_2021_985_MOESM1_ESM/Figure S4_ESM.pdf]

**(a)**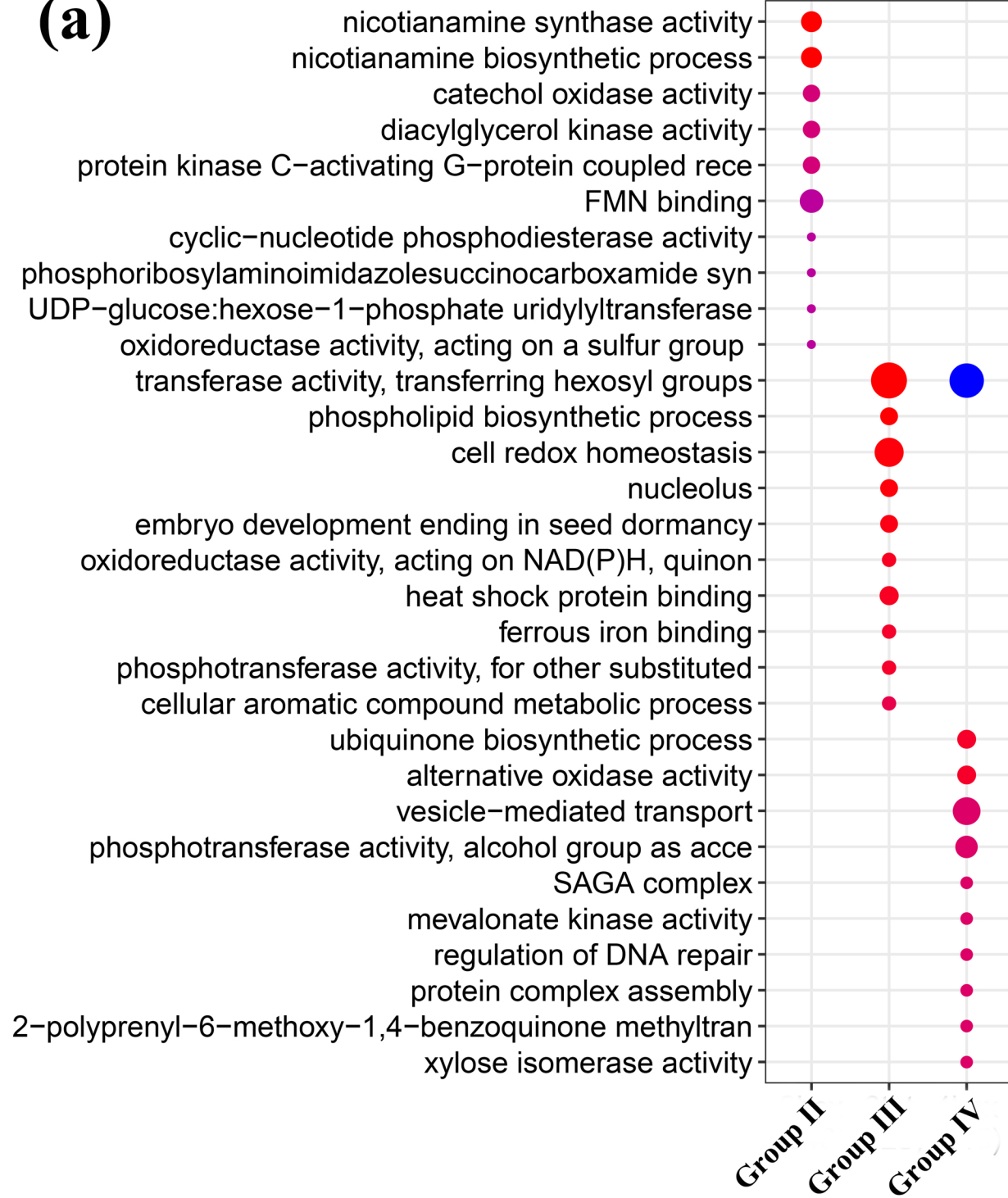**(b)**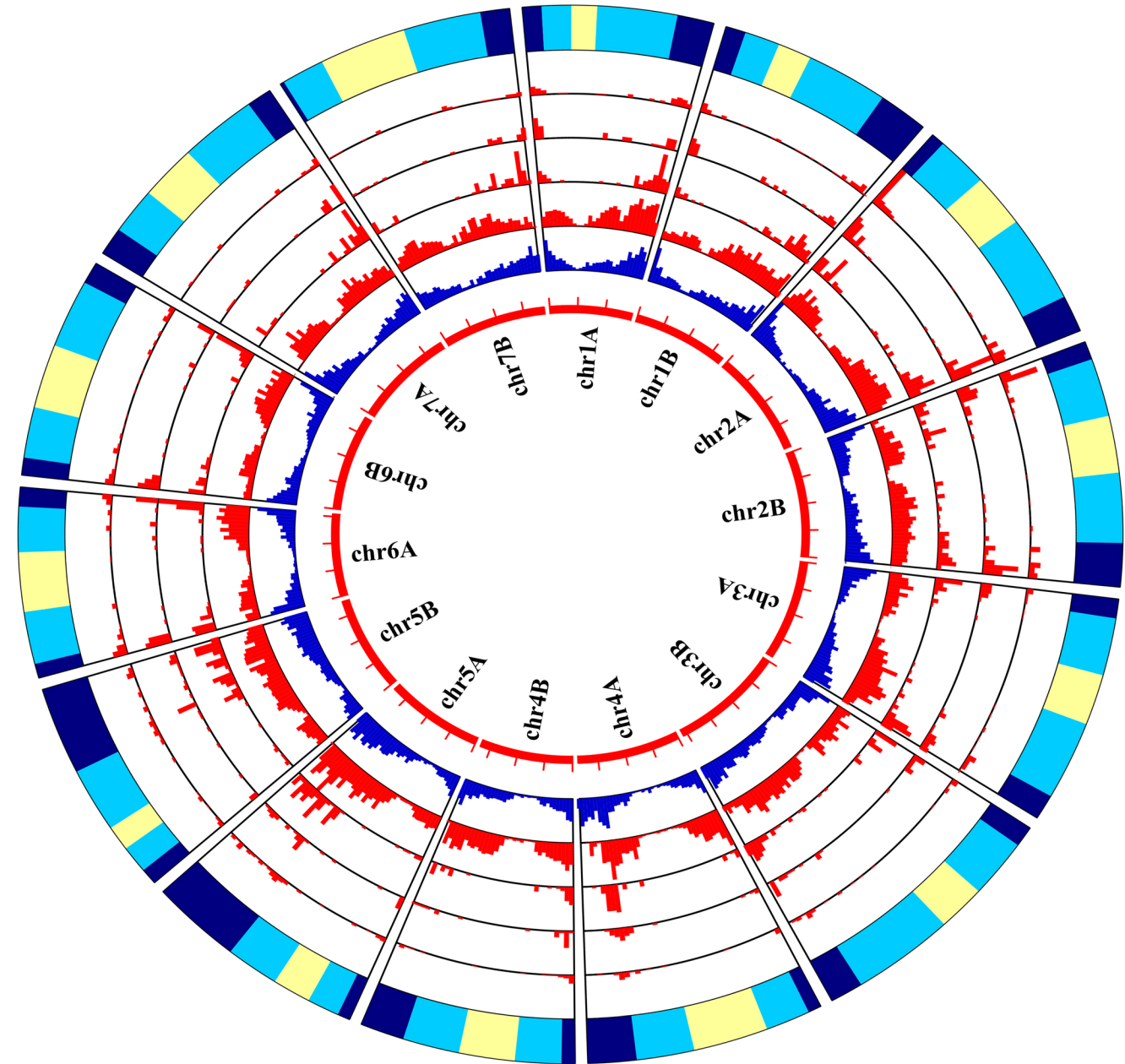

Supplement: Supplementary file 1 — Additional file 1: Figure S1. The relative proportions of raw data mapped to the three subgenomes of hexaploid wheat. (a), (b) and (c) are the H3K4me3 ChIP-seq data, H3K27me3 ChIP-seq data and RNA-seq data, respectively. Figure S2. Visualized correlation coefficients between the two biological replicates of the ChIP-seq data for the two histone markers and peaks of H3K4me3 and H3K27me3 at representative triad genes. (a) AABB components in H3K4me3; (b) Subgenome A in H3K4me3; (c) Subgenome B in H3K4me3; (d) AABB components in H3K27me3; (e) Subgenome A in H3K27me3; (f) Subgenome B in H3K27me3; (g) peaks of H3K4me3 in subgenomes A, B and D (from left to right in each panel); (h) peaks of H3K27me3 in subgenomes A, B and D (from left to right in each panel). Figure S3. Genome and chromosomal distribution of H3K27me3 in all the plant lines used in this study. Denotations are the same as in legend to Fig. 2. Figure S4. Examples of conservation and remodeling of the two histone modifications (H3K4me3 and H3K27me3) shown by integrative genomics viewer (IGV) snapshots. (a) Groups I-IV H3K4me3 histone modification patterns during the WTW → DTW → ETW process. For Group I (conserved), the three possible relationships, namely, A = B, A < B and A > B, in each of the WTW → DTW → ETW steps are presented; Group II shows changes that occurred in DTW and maintained in ETW; Group III shows changes that occurred in DTW and changed further in ETW; Group IV shows changes that only occurred in ETW. (b) Groups I-IV histone modification patterns (H3K27me3) in the TAA10 → ETW → XX329 ploidy transition process. For Pattern I (conserved), all three changing patterns, namely, A = B, A < B and A > B, in each of the TAA10 → ETW → XX329 steps are presented; Pattern II indicates reversible changes that are exclusively dependent on the presence of DD subgenome; Pattern III indicates changes that only occurred in XX329, reflecting prompt trans-subgenome regulation mediated by the presence of a novel DD [file 12915_2021_985_MOESM1_ESM.zip › 12915_2021_985_MOESM1_ESM/Figure S5_ESM.pdf]

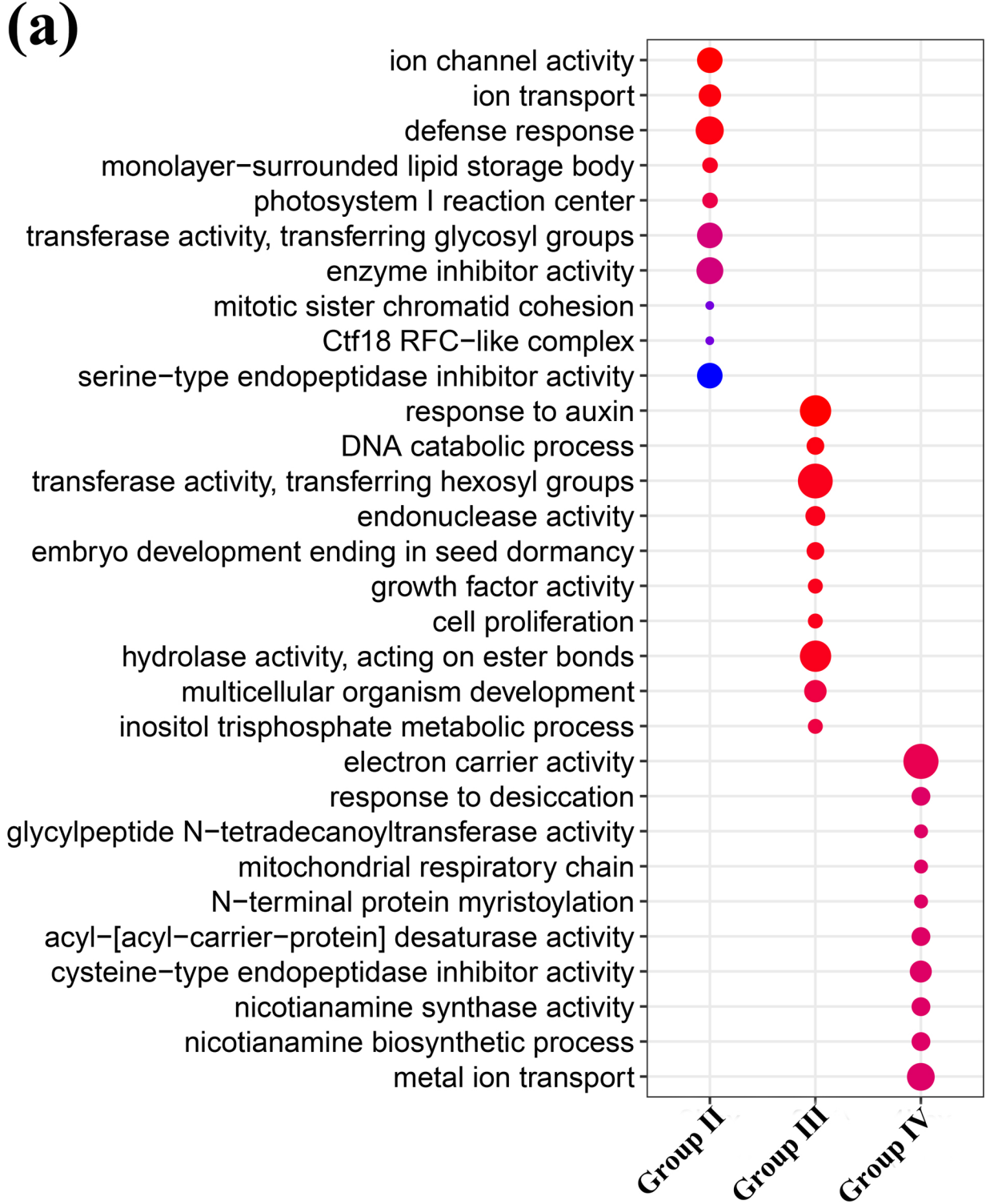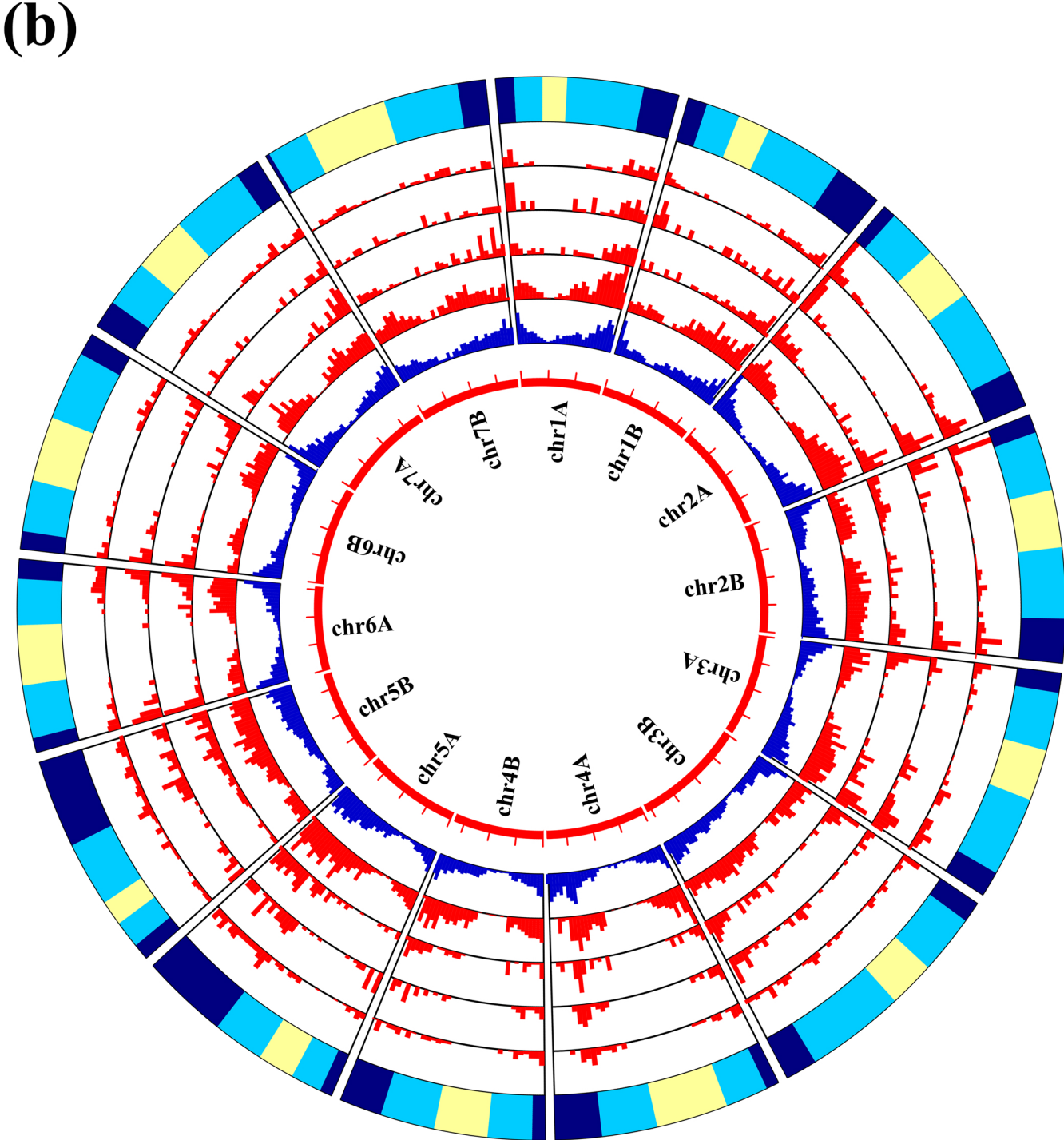

Supplement: Supplementary file 1 — Additional file 1: Figure S1. The relative proportions of raw data mapped to the three subgenomes of hexaploid wheat. (a), (b) and (c) are the H3K4me3 ChIP-seq data, H3K27me3 ChIP-seq data and RNA-seq data, respectively. Figure S2. Visualized correlation coefficients between the two biological replicates of the ChIP-seq data for the two histone markers and peaks of H3K4me3 and H3K27me3 at representative triad genes. (a) AABB components in H3K4me3; (b) Subgenome A in H3K4me3; (c) Subgenome B in H3K4me3; (d) AABB components in H3K27me3; (e) Subgenome A in H3K27me3; (f) Subgenome B in H3K27me3; (g) peaks of H3K4me3 in subgenomes A, B and D (from left to right in each panel); (h) peaks of H3K27me3 in subgenomes A, B and D (from left to right in each panel). Figure S3. Genome and chromosomal distribution of H3K27me3 in all the plant lines used in this study. Denotations are the same as in legend to Fig. 2. Figure S4. Examples of conservation and remodeling of the two histone modifications (H3K4me3 and H3K27me3) shown by integrative genomics viewer (IGV) snapshots. (a) Groups I-IV H3K4me3 histone modification patterns during the WTW → DTW → ETW process. For Group I (conserved), the three possible relationships, namely, A = B, A < B and A > B, in each of the WTW → DTW → ETW steps are presented; Group II shows changes that occurred in DTW and maintained in ETW; Group III shows changes that occurred in DTW and changed further in ETW; Group IV shows changes that only occurred in ETW. (b) Groups I-IV histone modification patterns (H3K27me3) in the TAA10 → ETW → XX329 ploidy transition process. For Pattern I (conserved), all three changing patterns, namely, A = B, A < B and A > B, in each of the TAA10 → ETW → XX329 steps are presented; Pattern II indicates reversible changes that are exclusively dependent on the presence of DD subgenome; Pattern III indicates changes that only occurred in XX329, reflecting prompt trans-subgenome regulation mediated by the presence of a novel DD [file 12915_2021_985_MOESM1_ESM.zip › 12915_2021_985_MOESM1_ESM/Figure S6_ESM.pdf]

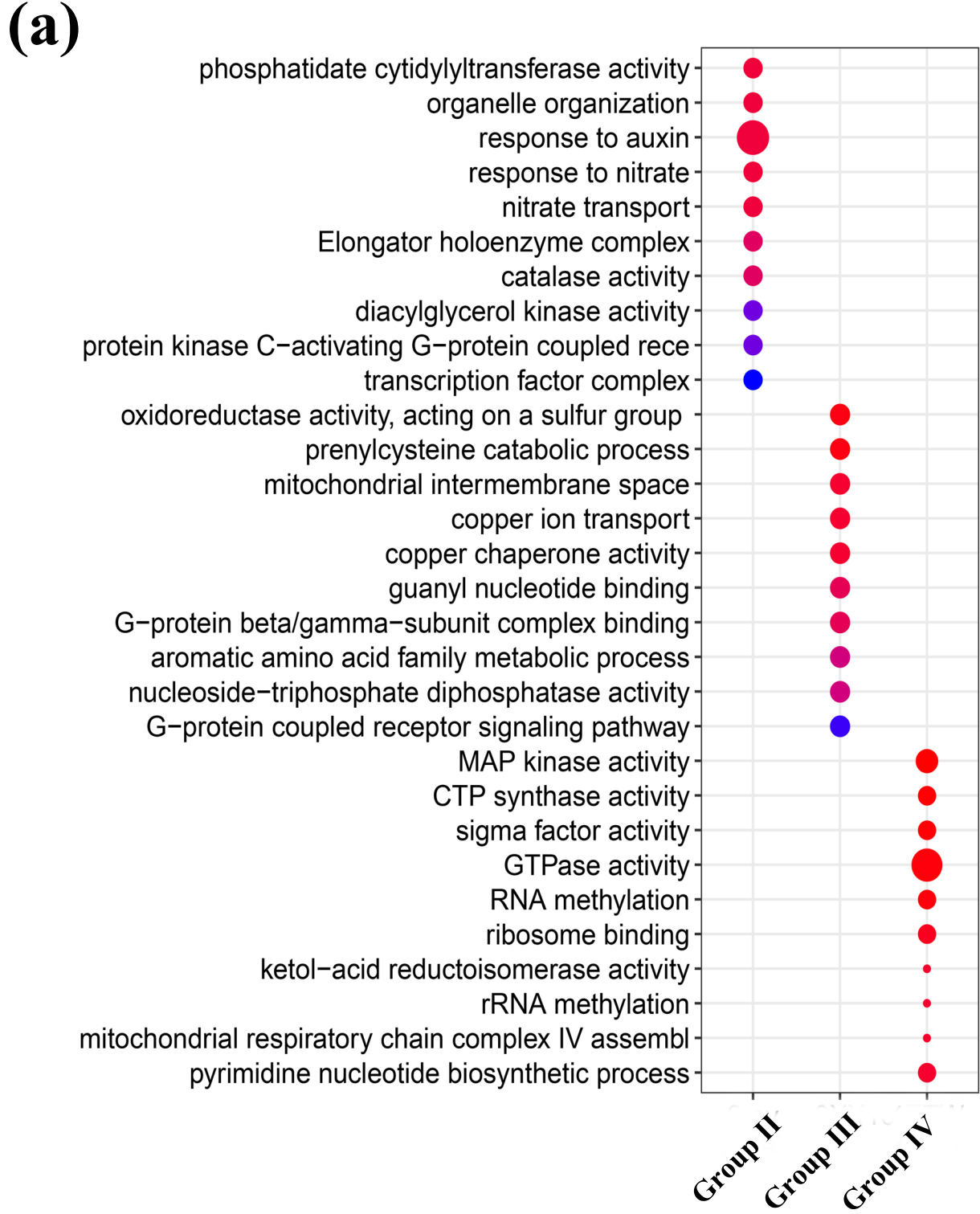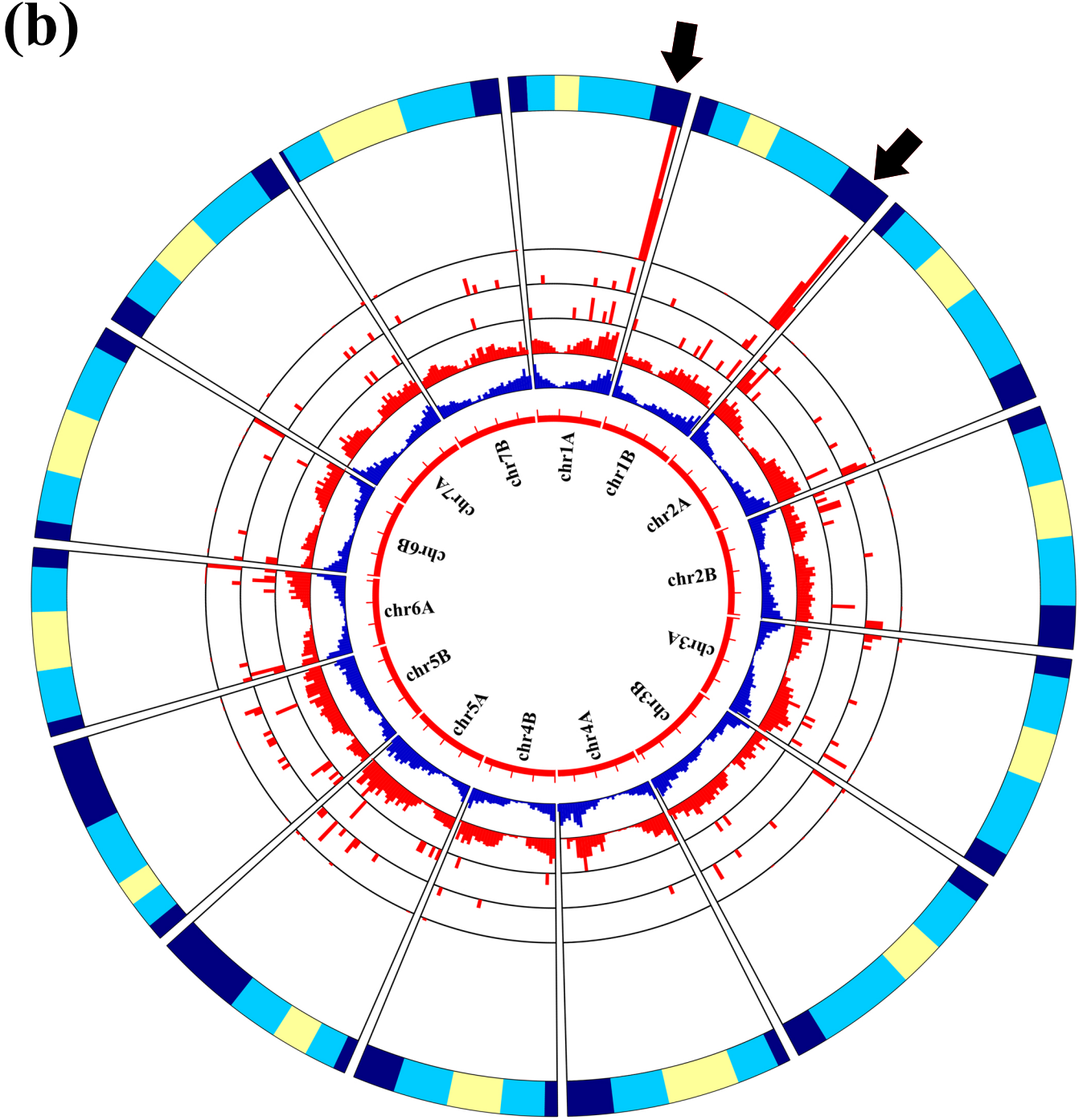

Supplement: Supplementary file 1 — Additional file 1: Figure S1. The relative proportions of raw data mapped to the three subgenomes of hexaploid wheat. (a), (b) and (c) are the H3K4me3 ChIP-seq data, H3K27me3 ChIP-seq data and RNA-seq data, respectively. Figure S2. Visualized correlation coefficients between the two biological replicates of the ChIP-seq data for the two histone markers and peaks of H3K4me3 and H3K27me3 at representative triad genes. (a) AABB components in H3K4me3; (b) Subgenome A in H3K4me3; (c) Subgenome B in H3K4me3; (d) AABB components in H3K27me3; (e) Subgenome A in H3K27me3; (f) Subgenome B in H3K27me3; (g) peaks of H3K4me3 in subgenomes A, B and D (from left to right in each panel); (h) peaks of H3K27me3 in subgenomes A, B and D (from left to right in each panel). Figure S3. Genome and chromosomal distribution of H3K27me3 in all the plant lines used in this study. Denotations are the same as in legend to Fig. 2. Figure S4. Examples of conservation and remodeling of the two histone modifications (H3K4me3 and H3K27me3) shown by integrative genomics viewer (IGV) snapshots. (a) Groups I-IV H3K4me3 histone modification patterns during the WTW → DTW → ETW process. For Group I (conserved), the three possible relationships, namely, A = B, A < B and A > B, in each of the WTW → DTW → ETW steps are presented; Group II shows changes that occurred in DTW and maintained in ETW; Group III shows changes that occurred in DTW and changed further in ETW; Group IV shows changes that only occurred in ETW. (b) Groups I-IV histone modification patterns (H3K27me3) in the TAA10 → ETW → XX329 ploidy transition process. For Pattern I (conserved), all three changing patterns, namely, A = B, A < B and A > B, in each of the TAA10 → ETW → XX329 steps are presented; Pattern II indicates reversible changes that are exclusively dependent on the presence of DD subgenome; Pattern III indicates changes that only occurred in XX329, reflecting prompt trans-subgenome regulation mediated by the presence of a novel DD [file 12915_2021_985_MOESM1_ESM.zip › 12915_2021_985_MOESM1_ESM/Figure S7_ESM.pdf]

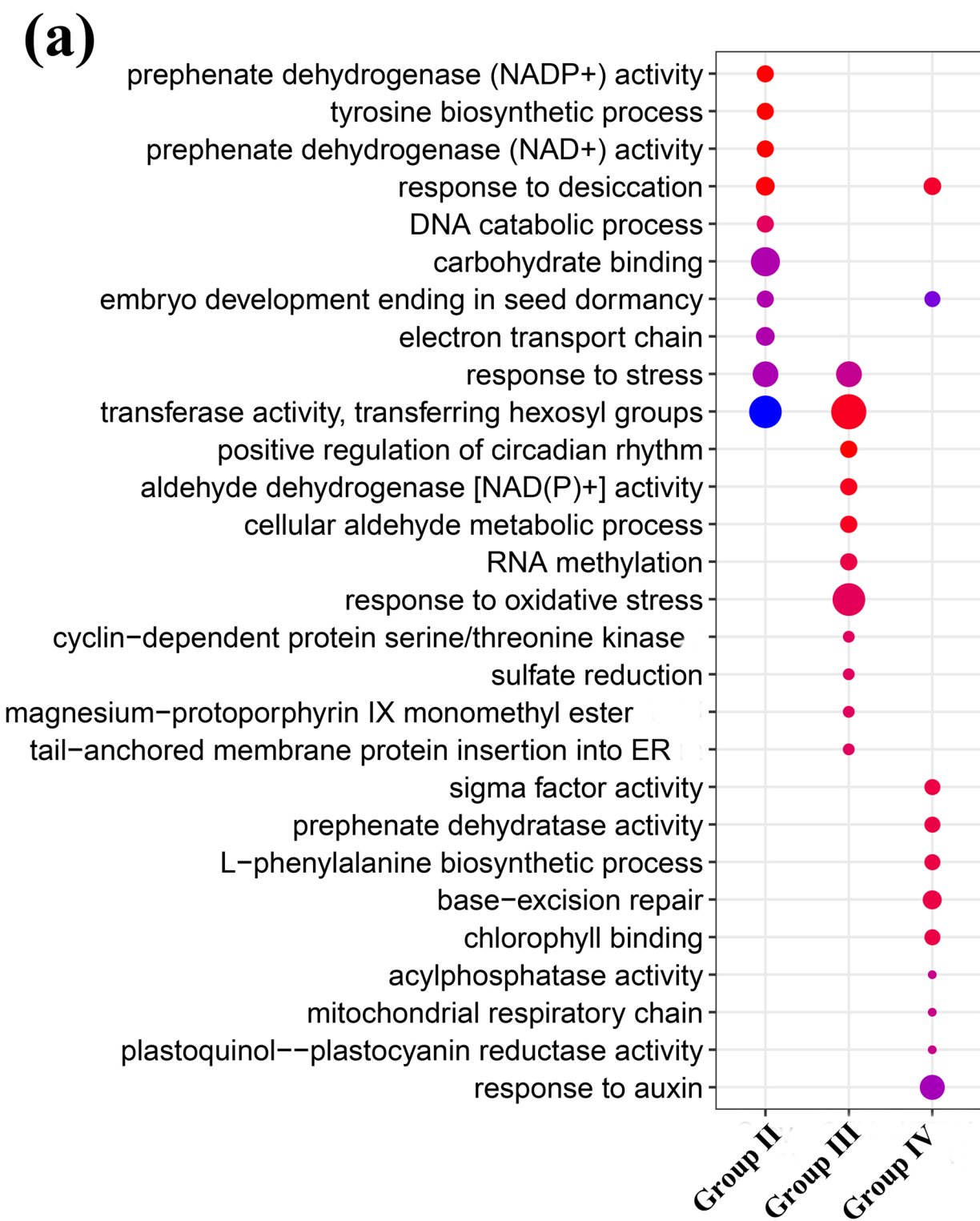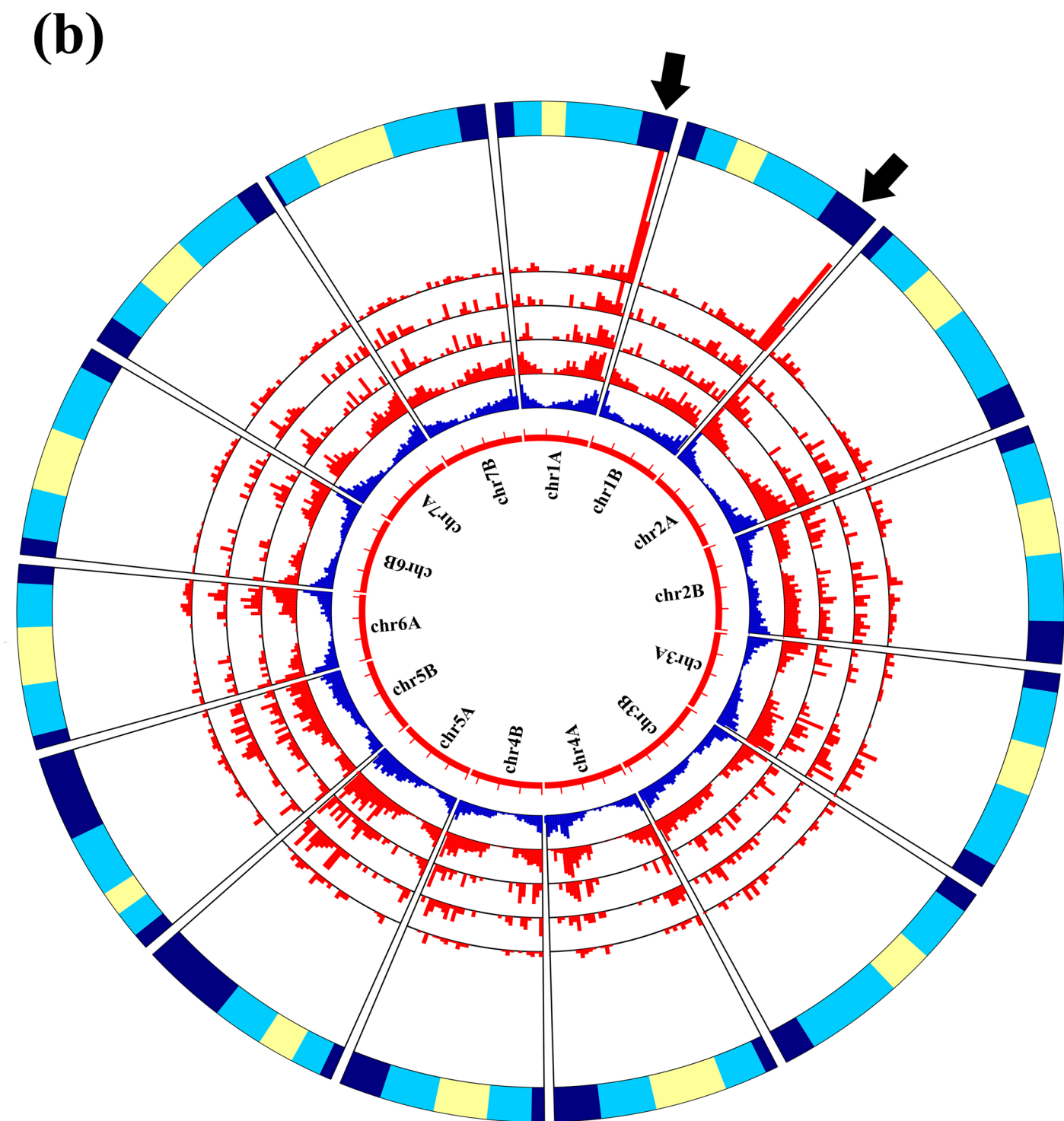

Supplement: Supplementary file 1 — Additional file 1: Figure S1. The relative proportions of raw data mapped to the three subgenomes of hexaploid wheat. (a), (b) and (c) are the H3K4me3 ChIP-seq data, H3K27me3 ChIP-seq data and RNA-seq data, respectively. Figure S2. Visualized correlation coefficients between the two biological replicates of the ChIP-seq data for the two histone markers and peaks of H3K4me3 and H3K27me3 at representative triad genes. (a) AABB components in H3K4me3; (b) Subgenome A in H3K4me3; (c) Subgenome B in H3K4me3; (d) AABB components in H3K27me3; (e) Subgenome A in H3K27me3; (f) Subgenome B in H3K27me3; (g) peaks of H3K4me3 in subgenomes A, B and D (from left to right in each panel); (h) peaks of H3K27me3 in subgenomes A, B and D (from left to right in each panel). Figure S3. Genome and chromosomal distribution of H3K27me3 in all the plant lines used in this study. Denotations are the same as in legend to Fig. 2. Figure S4. Examples of conservation and remodeling of the two histone modifications (H3K4me3 and H3K27me3) shown by integrative genomics viewer (IGV) snapshots. (a) Groups I-IV H3K4me3 histone modification patterns during the WTW → DTW → ETW process. For Group I (conserved), the three possible relationships, namely, A = B, A < B and A > B, in each of the WTW → DTW → ETW steps are presented; Group II shows changes that occurred in DTW and maintained in ETW; Group III shows changes that occurred in DTW and changed further in ETW; Group IV shows changes that only occurred in ETW. (b) Groups I-IV histone modification patterns (H3K27me3) in the TAA10 → ETW → XX329 ploidy transition process. For Pattern I (conserved), all three changing patterns, namely, A = B, A < B and A > B, in each of the TAA10 → ETW → XX329 steps are presented; Pattern II indicates reversible changes that are exclusively dependent on the presence of DD subgenome; Pattern III indicates changes that only occurred in XX329, reflecting prompt trans-subgenome regulation mediated by the presence of a novel DD [file 12915_2021_985_MOESM1_ESM.zip › 12915_2021_985_MOESM1_ESM/Figure S8_ESM.pdf]

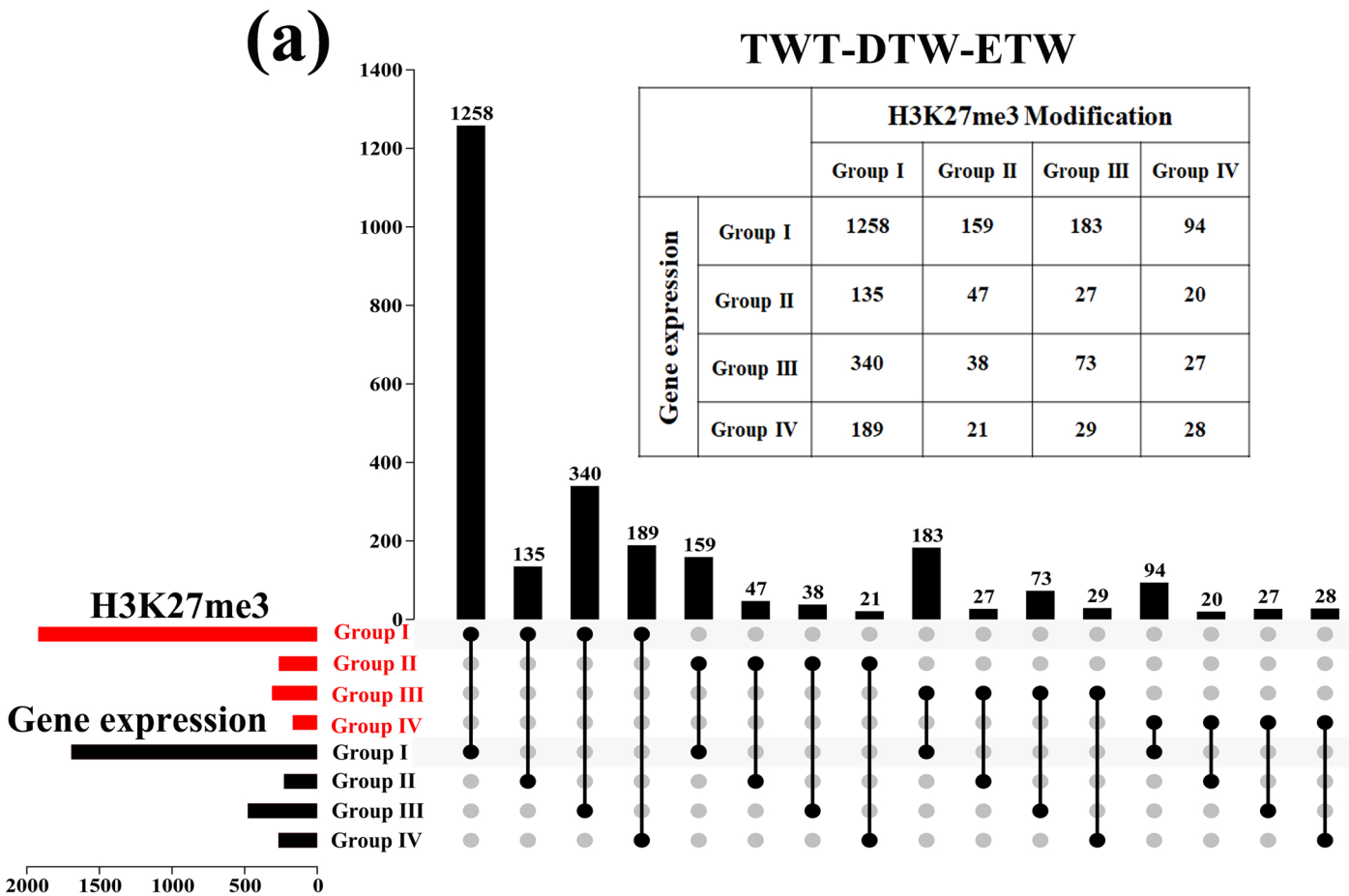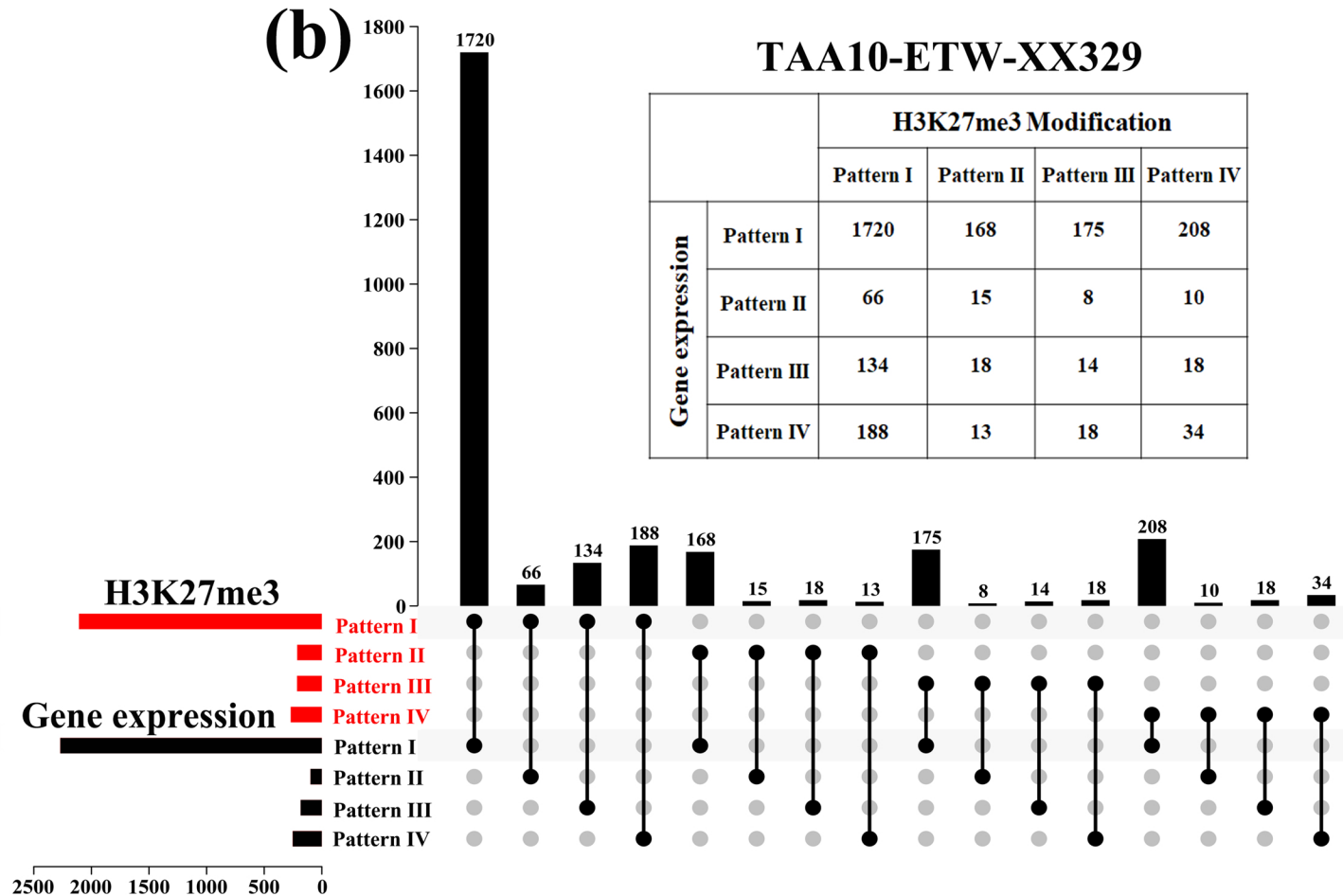

Supplement: Supplementary file 1 — Additional file 1: Figure S1. The relative proportions of raw data mapped to the three subgenomes of hexaploid wheat. (a), (b) and (c) are the H3K4me3 ChIP-seq data, H3K27me3 ChIP-seq data and RNA-seq data, respectively. Figure S2. Visualized correlation coefficients between the two biological replicates of the ChIP-seq data for the two histone markers and peaks of H3K4me3 and H3K27me3 at representative triad genes. (a) AABB components in H3K4me3; (b) Subgenome A in H3K4me3; (c) Subgenome B in H3K4me3; (d) AABB components in H3K27me3; (e) Subgenome A in H3K27me3; (f) Subgenome B in H3K27me3; (g) peaks of H3K4me3 in subgenomes A, B and D (from left to right in each panel); (h) peaks of H3K27me3 in subgenomes A, B and D (from left to right in each panel). Figure S3. Genome and chromosomal distribution of H3K27me3 in all the plant lines used in this study. Denotations are the same as in legend to Fig. 2. Figure S4. Examples of conservation and remodeling of the two histone modifications (H3K4me3 and H3K27me3) shown by integrative genomics viewer (IGV) snapshots. (a) Groups I-IV H3K4me3 histone modification patterns during the WTW → DTW → ETW process. For Group I (conserved), the three possible relationships, namely, A = B, A < B and A > B, in each of the WTW → DTW → ETW steps are presented; Group II shows changes that occurred in DTW and maintained in ETW; Group III shows changes that occurred in DTW and changed further in ETW; Group IV shows changes that only occurred in ETW. (b) Groups I-IV histone modification patterns (H3K27me3) in the TAA10 → ETW → XX329 ploidy transition process. For Pattern I (conserved), all three changing patterns, namely, A = B, A < B and A > B, in each of the TAA10 → ETW → XX329 steps are presented; Pattern II indicates reversible changes that are exclusively dependent on the presence of DD subgenome; Pattern III indicates changes that only occurred in XX329, reflecting prompt trans-subgenome regulation mediated by the presence of a novel DD [file 12915_2021_985_MOESM1_ESM.zip › 12915_2021_985_MOESM1_ESM/Figure S9_ESM.pdf]
