# Supplementary material for: Conservation and trans-regulation of histone modification in the A and B subgenomes of polyploid wheat during domestication and ploidy transition
Source: BMC Biol. 2021 Mar 9;19:42. doi: 10.1186/s12915-021-00985-7 (PMC7944620; doi:10.1186/s12915-021-00985-7)
Supplement: Supplementary file 2 — Additional file 2: Table S1. Information related to data mapping for ChIP-seq and RNA-Seq. [file 12915_2021_985_MOESM2_ESM.docx]

Table S1 Information of data mapping for ChIP-Seq and RNA-Seq

|  | **Samples** | **raw reads** | **mapping rate** | **unique mapped reads** | **mapping rate** |
| --- | --- | --- | --- | --- | --- |
| **H3K4me3** | ETW_k4_1 | 50749078 | 80.28% | 29602980 | 58.33% |
|  | ETW_k4_2 | 54761098 | 83.03% | 34881647 | 63.70% |
|  | TAA10_k4_1 | 70920778 | 81.35% | 43435026 | 61.24% |
|  | TAA10_k4_2 | 74732005 | 84.88% | 50615750 | 67.73% |
|  | TD265_k4_1 | 55503469 | 71.46% | 26218823 | 47.24% |
|  | TD265_k4_2 | 53803280 | 74.30% | 27081789 | 50.33% |
|  | TQ18_k4_1 | 33029942 | 80.96% | 19180015 | 58.07% |
|  | TQ18_k4_2 | 32192248 | 85.00% | 21015404 | 65.28% |
|  | TTR13_k4_1 | 52264108 | 75.79% | 27320935 | 52.27% |
|  | TTR13_k4_2 | 58664927 | 79.34% | 33797396 | 57.61% |
|  | XX329_k4_1 | 72463922 | 80.87% | 42854863 | 59.14% |
|  | XX329_k4_2 | 70719822 | 84.73% | 47427440 | 67.06% |

|  | **Samples** | **raw reads** | **mapping rate** | **unique mapped reads** | **mapping rate** |
| --- | --- | --- | --- | --- | --- |
| **H3K27me3** | ETW_k27_1 | 58110313 | 68.72% | 28003768 | 48.19% |
|  | ETW_k27_2 | 56044850 | 69.96% | 27892822 | 49.77% |
|  | TAA10_k27_1 | 86215393 | 58.15% | 36145740 | 41.92% |
|  | TAA10_k27_2 | 73648484 | 60.92% | 32888809 | 44.66% |
|  | TD265_k27_1 | 53877896 | 65.03% | 20601065 | 38.24% |
|  | TD265_k27_2 | 54043045 | 65.73% | 20618563 | 38.15% |
|  | TTR13_k27_1 | 52465576 | 68.06% | 23693989 | 45.16% |
|  | TTR13_k27_2 | 52316684 | 70.34% | 24990726 | 47.77% |
|  | TQ18_k27_1 | 32979790 | 68.87% | 15194658 | 46.07% |
|  | TQ18_k27_2 | 35871594 | 73.28% | 18764350 | 52.31% |
|  | XX329_k27_1 | 81828061 | 71.28% | 40807888 | 49.87% |
|  | XX329_k27_2 | 74474215 | 71.65% | 37410445 | 50.23% |

|  | **Samples** | **raw reads** | **mapping rate** | **unique mapped reads** | **mapping rate** |
| --- | --- | --- | --- | --- | --- |
| transcriptome | ETW_transcriptome_1 | 48699574 | 88.25% | 27051833 | 55.55% |
|  | ETW_transcriptome_2 | 40601776 | 89.45% | 20564326 | 50.65% |
|  | TAA10_transcriptome_1 | 49310513 | 88.25% | 25522276 | 51.76% |
|  | TAA10_transcriptome_2 | 49638775 | 89.11% | 26308133 | 53.00% |
|  | TD265_transcriptome_1 | 40126447 | 87.33% | 24762407 | 61.71% |
|  | TD265_transcriptome_2 | 43091962 | 86.96% | 26853485 | 62.32% |
|  | TD265_transcriptome_3 | 33418164 | 86.64% | 20517239 | 61.40% |
|  | TTR13_transcriptome_1 | 49632210 | 89.90% | 26439502 | 53.27% |
|  | TTR13_transcriptome_2 | 48940069 | 90.23% | 27157351 | 55.49% |
|  | TQ18_transcriptome_1 | 48229978 | 88.70% | 26026096 | 53.96% |
|  | TQ18_transcriptome_2 | 50975011 | 87.40% | 26584919 | 52.15% |
|  | XX329_transcriptome_1 | 57962977 | 89.51% | 30522037 | 52.66% |
|  | XX329_transcriptome_2 | 50985813 | 88.48% | 25069635 | 49.17% |
