# Supplementary material for: Conservation and trans-regulation of histone modification in the A and B subgenomes of polyploid wheat during domestication and ploidy transition
Source: BMC Biol. 2021 Mar 9;19:42. doi: 10.1186/s12915-021-00985-7 (PMC7944620; doi:10.1186/s12915-021-00985-7)
Supplement: Supplementary file 3 — Additional file 3: Table S2. The number of genes analyzed in wild, domesticated and extracted tetraploid wheat. [file 12915_2021_985_MOESM3_ESM.docx]

Table S2. The number of genes analyzed in wild, domesticated and extracted tetraploid wheat.

|  | **H3K4me3** | | | **H3K27me3** | | | **Transcriptome** | | |
| --- | --- | --- | --- | --- | --- | --- | --- | --- | --- |
|  | **TD265** | **TTR13** | **ETW** | **TD265** | **TTR13** | **ETW** | **TD265** | **TTR13** | **ETW** |
| **A=B** | **12,921 (89.4%)** | **13,069 (90.5%)** | **13250 (91.7%)** | **7,603 (84.9%)** | **7,595 (84.8%)** | **7,339 (81.9%)** | **7,084 (80.5%)** | **6,703 (76.3%)** | **6,869 (78.1%)** |
| **A>B** | **996**  **(6.9%)** | **826**  **(5.7%)** | **767**  **(5.3%)** | **792**  **(8.8%)** | **757**  **(8.5%)** | **806**  **(9.0%)** | **1,007 (11.4%)** | **1,147 (13.1%)** | **1,069 (12.2%)** |
| **A<B** | **531**  **(3.7%)** | **552**  **(3.8%)** | **431**  **(3.0%)** | **565**  **(6.3%)** | **606**  **(6.8%)** | **815**  **(9.1%)** | **712 (8.1%)** | **938 (10.7%)** | **858 (9.8%)** |
| **total** | **14,448** | **14,447** | **14,448** | **8,960** | **8,958** | **8,960** | **8,803** | **8,788** | **8,796** |
| **Binom.test** | **996 *vs* 531**  ***p* value <**  **2.2e-16** | **826 *vs* 552**  ***p* value = 1.605e-13** | **767 *vs* 431**  ***p* value < 2.2e-16** | **792 *vs* 565**  ***p* value = 7.8e-10** | **757 *vs* 606**  ***p* value = 4.766e-5** | **806 *vs* 815**  ***p* value = 0.843** | **1,007 *vs* 712**  ***p* value = 1.18e-12** | **1,147 *vs* 938**  ***p* value = 5.14e-6** | **1,069 *vs* 858**  ***p* value = 1.68e-6** |

The modification patterns of the A=B，A>B，A<B genes that analyzed in the three tetraploid wheats, dicoccoides (line TD265), durum (cv. TTR13) and extracted-tetraploid wheat (ETW). The pattern of A>B was significantly higher than A<B based on Binom.test in the three tetraploid wheats.
