# Supplementary material for: Conservation and trans-regulation of histone modification in the A and B subgenomes of polyploid wheat during domestication and ploidy transition
Source: BMC Biol. 2021 Mar 9;19:42. doi: 10.1186/s12915-021-00985-7 (PMC7944620; doi:10.1186/s12915-021-00985-7)
Supplement: Supplementary file 4 — Additional file 4: Table S3. Pairwise comparison of three modes of histone modification in wild, domesticated and extracted tetraploid wheat. [file 12915_2021_985_MOESM4_ESM.docx]

| H3K4me3 | A = B | A > B | A < B |
| --- | --- | --- | --- |
| TD265 VS TTR13 | p value = 0.004036 | p value = 4.308e-05 | p value = 0.5356 |
| TD265 VS ETW | p value = 4.047e-11 | p value = 2.098e-08 | p value = 0.001169 |
| TTR13 VS ETW | p value = 0.0002029 | p value = 0.1349 | p value = 9.852e-05 |

Table S3. Pairwise comparison of three modes of histone modification in wild, domesticated and extracted tetraploid wheat.

| H3K27me3 | A = B | A > B | A < B |
| --- | --- | --- | --- |
| TD265 VS TTR13 | p value = 0.8842 | p value = 0.3661 | p value = 0.2266 |
| TD265 VS ETW | p value = 1.307e-07 | p value = 0.7333 | p value = 3.018e-12 |
| TTR13 VS ETW | p value = 3.191e-07 | p value = 0.2038 | p value = 8.897e-09 |
