# Supplementary material for: Conservation and trans-regulation of histone modification in the A and B subgenomes of polyploid wheat during domestication and ploidy transition
Source: BMC Biol. 2021 Mar 9;19:42. doi: 10.1186/s12915-021-00985-7 (PMC7944620; doi:10.1186/s12915-021-00985-7)
Supplement: Supplementary file 5 — Additional file 5: Table S4. The triad genes analyzed in wild, domesticated and extracted tetraploid wheat. [file 12915_2021_985_MOESM5_ESM.docx]

Table S4. The triads genes analyzed in wild, domesticated and extracted tetraploid wheat.

|  |  |  |  | **H3K4me3** | | **H3K27me3** | | **transcriptome** | |
| --- | --- | --- | --- | --- | --- | --- | --- | --- | --- |
|  | **TD265_type** | **TTR13_type** | **ETW_type** | **Number**  **(ratio)** | **total** | **Number (ratio)** | **total** | **Number**  **(ratio)** | **total** |
| **Group I** | **A=B** | **A=B** | **A=B** | **12179 (84.3%)** | **88.3%** | **6074 (67.8%)** | **70.9%** | **5730 (65.3%)** | \| **74.3%** \| \| --- \| \| \| |
|  | **A<B** | **A<B** | **A<B** | **228 (1.6%)** |  | **112 (1.3%)** |  | **335 (3.8%)** |  |
|  | **A>B** | **A>B** | **A>B** | **349 (2.4%)** |  | **159 (1.8%)** |  | **450 (5.1%)** |  |
| **Group II-III** | **A<B** | **A=B** | **A=B, A<B, A>B** | **163 (1.1%)** | **7.8%** | **324 (3.6%)** | **18.1%** | **237 (2.7%)** | \| **18.5%** \| \| --- \| \| \| \| |
|  | **A=B** | **A<B** | **A=B, A<B, A>B** | **192 (1.3%)** |  | **377 (4.2%)** |  | **456 (5.2%)** |  |
|  | **A=B** | **A>B** | **A=B, A<B, A>B** | **297 (2.1%)** |  | **438 (4.9%)** |  | **545 (6.2%)** |  |
|  | **A>B** | **A=B** | **A=B, A<B, A>B** | **474 (3.3%)** |  | **484 (5.4%)** |  | **386 (4.4%)** |  |
| **Group IV** | **A<B** | **A<B** | **A=B** | **106 (0.7%)** | **3.6%** | **74 (0.8%)** | **10.0%** | **82 (0.9%)** | \| **6.2%** \| \| --- \| \| \| \| |
|  | **A=B** | **A=B** | **A<B** | **84 (0.6%)** |  | **407 (4.5%)** |  | **160 (1.8%)** |  |
|  | **A=B** | **A=B** | **A>B** | **169 (1.2%)** |  | **304 (3.4%)** |  | **185 (2.1%)** |  |
|  | **A>B** | **A>B** | **A=B** | **155 (1.1%)** |  | **109 (1.2%)** |  | **113 (1.3%)** |  |
| **Ambiguous** |  |  |  | **51 (0.4%)** | **0.4%** | **91 (1.0%)** | **1.0%** | **92 (1.0%)** | **1.0%** |

The evolution and domestication at hexaploid level had different influences and forces on the epigenetic modifications and gene expression between the subgenomes compared to the evolution at the tetraploid level.
