# Supplementary material for: Conservation and trans-regulation of histone modification in the A and B subgenomes of polyploid wheat during domestication and ploidy transition
Source: BMC Biol. 2021 Mar 9;19:42. doi: 10.1186/s12915-021-00985-7 (PMC7944620; doi:10.1186/s12915-021-00985-7)
Supplement: Supplementary file 8 — Additional file 8: Table S7. The transformation of genes analyzed in the hexaploidy-extracted tetraploid – resynthesized hexaploidy, TAA10 → ETW → XX329 ploidy transition process. [file 12915_2021_985_MOESM8_ESM.docx]

Table S7. The transformation of genes analyzed in the hexaploidy-extracted tetraploid –resynthesised hexaploidy, TAA10-ETW-XX329 ploidy transition process.

|  |  |  |  | **H3K4me3** | | **H3K27me3** | | **transcriptome** | |
| --- | --- | --- | --- | --- | --- | --- | --- | --- | --- |
|  | **TAA10_type** | **ETW_type** | **XX329_type** | **Number**  **(ratio)** | **total** | **Number (ratio)** | **total** | **Number**  **(ratio)** | **total** |
| **Pattern I** | **A=B** | **A=B** | **A=B** | **12858 (89.1%)** | **96.1%** | **5922 (66.3%)** | **76.7%** | **6215 (70.9%)** | \| **87.1%** \| \| --- \| \| \| |
|  | **A<B** | **A<B** | **A<B** | **373 (2.6%)** |  | **404 (4.5%)** |  | **645 (7.4%)** |  |
|  | **A>B** | **A>B** | **A>B** | **659 (4.6%)** |  | **536 (6.0%)** |  | **782 (8.9%)** |  |
| **Pattern II** | **A<B** | **A=B** | **A<B** | **33 (0.2%)** | **0.9%** | **94 (1.1%)** | \| **7.0%** \| \| --- \|     **7.0%** | **57 (0.7%)** | \| **2.5%** \| \| --- \| \| \| \| |
|  | **A=B** | **A<B** | **A=B** | **24 (0.2%)** |  | **223 (2.5%)** |  | **53 (0.6%)** |  |
|  | **A=B** | **A>B** | **A=B** | **43 (0.3%)** |  | **112 (1.3%)** |  | **69 (0.8%)** |  |
|  | **A>B** | **A=B** | **A>B** | **36 (0.2%)** |  | **198 (2.2%)** |  | **42 (0.5%)** |  |
| **Pattern III** | **A<B** | **A<B** | **A=B** | **16 (0.1%)** | **0.9%** | **73 (0.8%)** | \| **7.0%** \| \| --- \|     **7.0%** | **50 (0.6%)** | \| **4.1%** \| \| --- \| \| \| \| |
|  | **A=B** | **A=B** | **A<B** | **44 (0.3%)** |  | **178 (2.0%)** |  | **130 (1.5%)** |  |
|  | **A=B** | **A=B** | **A>B** | **58 (0.4%)** |  | **314 (3.5%)** |  | **112 (1.3%)** |  |
|  | **A>B** | **A>B** | **A=B** | **16 (0.1%)** |  | **51 (0.6%)** |  | **70 (0.8%)** |  |
| **Pattern IV** | **A<B** | **A=B** | **A=B** | **192 (1.3%)** | **2.0%** | **303 (3.4%)** | **8.6%** | **198 (2.3%)** | **6.1%** |
|  | **A=B** | **A<B** | **A<B** | **18 (0.1%)** |  | **86 (1.0%)** |  | **98 (1.1%)** |  |
|  | **A=B** | **A>B** | **A>B** | **37 (0.3%)** |  | **71 (0.8%)** |  | **123 (1.4%)** |  |
|  | **A>B** | **A=B** | **A=B** | **27 (0.2%)** |  | **295 (3.3%)** |  | **106 (1.2%)** |  |
| **Ambiguous** |  |  |  | **3 (0.0%)** | **0.0%** | **67 (0.7%)** | **0.7%** | **14 (0.2%)** | **0.2%** |
