# Supplementary material for: Conservation and trans-regulation of histone modification in the A and B subgenomes of polyploid wheat during domestication and ploidy transition
Source: BMC Biol. 2021 Mar 9;19:42. doi: 10.1186/s12915-021-00985-7 (PMC7944620; doi:10.1186/s12915-021-00985-7)
Supplement: Supplementary file 11 — Additional file 11: Table S10. Gene Ontology (GO) enrichment terms and genes used for GO analysis of H3K27me3 modification in TAA10 → ETW → XX329 ploidy transition process. [file 12915_2021_985_MOESM11_ESM.docx]

Table S10. The pairwise wilcox test analyzed in the hexaploidy-extracted tetraploid –resynthesised hexaploidy, TAA10-ETW-XX329 ploidy transition process.

| marker |  | flag_new_type | TAA10_ETW_AA | ETW_XX329_AA | TAA10_XX329_AA | TAA10_ETW_BB | ETW_XX329_BB | TAA10_XX329_BB | TAA10_XX329_DD | TD265_ETW_AA | TTR13_ETW_AA | TD265_TTR13_AA | TD265_ETW_BB | TTR13_ETW_BB | TD265_TTR13_BB |
| --- | --- | --- | --- | --- | --- | --- | --- | --- | --- | --- | --- | --- | --- | --- | --- |
| H3K4me3 | Pattern I | A=B→A=B→A=B | < 2.2e-16 | < 2.2e-16 | 0.738106245 | < 2.2e-16 | < 2.2e-16 | 0.99740955 | 0.029946414 | 1.19E-16 | 5.03E-20 | 0.49122107 | 3.43E-34 | 2.43E-13 | 1.35E-06 |
|  |  | A>B→A>B→A>B | 1.09E-46 | 5.15E-43 | 0.584265504 | 3.09E-09 | 1.68E-11 | 0.292921534 | 0.293633096 | 2.40E-09 | 5.58E-07 | 0.315197045 | 1.03E-33 | 1.38E-20 | 0.008311389 |
|  |  | A<B→A<B→A<B | 3.73E-09 | 3.71E-10 | 0.66323612 | 5.88E-26 | 6.84E-24 | 0.646308537 | 0.781173838 | 2.83E-08 | 0.001917941 | 0.02672242 | 1.67E-06 | 0.000141329 | 0.309468321 |
|  | Pattern II | A=B→A>B→A=B | 0.00024514 | 0.000472665 | 0.889180288 | 0.280358414 | 0.299903568 | 0.998778259 | 0.986561452 | 0.006271205 | 0.027780591 | 0.40571365 | 0.535159074 | 0.097870874 | 0.036881579 |
|  |  | A=B→A<B→A=B | 0.499616438 | 0.237835678 | 0.6069888 | 0.005832397 | 0.004194446 | 0.790063925 | 0.322304168 | 0.381217764 | 0.852457261 | 0.550371288 | 0.181968134 | 0.229192983 | 0.812620696 |
|  |  | A>B→A=B→A>B | 0.011498545 | 0.015380752 | 0.812087499 | 2.11E-05 | 1.51E-05 | 0.966536 | 0.858879282 | 0.824507121 | 0.650194234 | 0.877745506 | 0.57454423 | 0.830733656 | 0.759865165 |
|  |  | A<B→A=B→A=B | 1.18E-05 | 5.36E-05 | 0.531455327 | 0.030809904 | 0.052250489 | 0.839505501 | 0.875233363 | 0.990921971 | 0.608647713 | 0.786549781 | 0.133717283 | 0.285861677 | 0.602292697 |
|  | Pattern III | A=B→A=B→A>B | 0.000142267 | 0.006756424 | 0.267735301 | 9.54E-05 | 2.42E-08 | 0.019597306 | 0.881030049 | 0.53707646 | 0.792447304 | 0.768424625 | 0.946187374 | 0.831898003 | 0.805291515 |
|  |  | A=B→A=B→A<B | 0.00091519 | 2.28E-06 | 0.065061392 | 0.002364156 | 0.010530146 | 0.541199428 | 0.154249456 | 0.622262263 | 0.474881222 | 0.272969468 | 0.82553356 | 0.987016108 | 0.853285956 |
|  |  | A>B→A>B→A=B | 0.006236141 | 0.004021803 | 0.713808073 | 0.029437353 | 0.187300553 | 0.205999989 | 0.973411315 | 0.654996034 | 0.815497201 | 0.419189951 | 0.962782363 | 0.526165732 | 0.654996034 |
|  |  | A<B→A<B→A=B | 0.027440684 | 0.693996149 | 0.051914062 | 0.022946055 | 0.037421803 | 0.90977241 | 0.360167319 | 0.128136264 | 0.464297814 | 0.489247397 | 0.281854848 | 0.526165732 | 0.815497201 |
|  | Pattern IV | A=B→A>B→A>B | 3.59E-05 | 0.000569476 | 0.285498641 | 0.093383406 | 0.001153379 | 0.115425284 | 0.816524255 | 0.931237812 | 0.697089105 | 0.778041077 | 0.244454985 | 0.556080259 | 0.79278337 |
|  |  | A=B→A<B→A<B | 0.034143393 | 0.002969701 | 0.284011125 | 0.000797613 | 0.002856973 | 0.422980387 | 0.968754792 | 0.512283856 | 0.602578795 | 0.378886189 | 0.063686072 | 0.343408108 | 0.397430905 |
|  |  | A>B→A=B→A=B | 0.009263371 | 0.007452628 | 0.642714653 | 9.83E-06 | 0.004937401 | 0.043541936 | 0.812996873 | 0.422638672 | 0.447947902 | 0.742360889 | 0.616530632 | 0.474096688 | 0.784523922 |
|  |  | A<B→A=B→A=B | 2.64E-119 | 2.19E-09 | 1.65E-114 | 1.78E-12 | 2.76E-13 | 0.691578032 | 3.43E-20 | 0.475898403 | 0.467085974 | 0.976253847 | 0.025607793 | 0.096414098 | 0.645875704 |
| H3K27me3 | Pattern I | A=B→A=B→A=B | 1.18E-105 | 1.86E-123 | 0.055926239 | 3.94E-154 | 1.38E-177 | 0.009876959 | 4.85E-05 | 2.60E-189 | 2.51E-115 | 3.20E-12 | 1.81E-133 | 1.01E-83 | 7.26E-09 |
|  |  | A>B→A>B→A>B | 7.33E-12 | 6.76E-10 | 0.57303683 | 5.58E-10 | 1.22E-14 | 0.077068498 | 0.05207626 | 9.55E-06 | 0.005237708 | 0.121855904 | 4.34E-78 | 9.36E-42 | 3.20E-08 |
|  |  | A<B→A<B→A<B | 7.91E-12 | 4.46E-17 | 0.086501738 | 1.46E-18 | 1.84E-19 | 0.541787155 | 0.007516596 | 3.13E-64 | 1.90E-30 | 5.03E-09 | 8.88E-12 | 2.94E-06 | 0.073858039 |
|  | Pattern II | A=B→A>B→A=B | 1.31E-10 | 8.70E-12 | 0.48007368 | 5.07E-06 | 0.003586032 | 0.073333491 | 0.15746605 | 0.00031477 | 0.050860643 | 0.105806733 | 1.54E-30 | 3.35E-26 | 0.01109545 |
|  |  | A=B→A<B→A=B | 0.000492254 | 0.023229387 | 0.201925762 | 2.10E-16 | 1.21E-18 | 0.399132169 | 0.156796617 | 2.16E-54 | 6.85E-44 | 0.002923708 | 0.001020496 | 0.008206373 | 0.660570158 |
|  |  | A>B→A=B→A>B | 0.766368497 | 0.180463044 | 0.257187692 | 4.91E-38 | 6.45E-43 | 0.191539445 | 0.189240004 | 1.46E-16 | 4.20E-12 | 0.41171932 | 1.81E-23 | 2.09E-09 | 1.02E-05 |
|  |  | A<B→A=B→A=B | 1.18E-31 | 3.15E-26 | 0.446835359 | 0.073712179 | 0.001213716 | 0.110757147 | 0.003357582 | 6.51E-16 | 7.93E-07 | 0.001090566 | 2.82E-08 | 0.00039263 | 0.128314392 |
|  | Pattern III | A=B→A=B→A>B | 7.83E-11 | 2.28E-05 | 0.017434753 | 1.29E-20 | 2.10E-66 | 3.38E-25 | 0.051411715 | 2.26E-41 | 5.01E-15 | 8.60E-08 | 2.47E-44 | 1.37E-22 | 6.09E-06 |
|  |  | A=B→A=B→A<B | 3.53E-12 | 6.31E-47 | 9.80E-25 | 1.40E-12 | 8.13E-07 | 0.015075346 | 0.009535801 | 4.82E-29 | 7.57E-10 | 2.74E-09 | 1.59E-19 | 3.03E-07 | 5.07E-05 |
|  |  | A>B→A>B→A=B | 0.003408373 | 4.48E-05 | 0.125994184 | 0.0302193 | 0.064975767 | 3.90E-05 | 0.577779587 | 0.040999686 | 0.155707925 | 0.684139733 | 5.30E-10 | 1.19E-11 | 0.774983659 |
|  |  | A<B→A<B→A=B | 0.005988676 | 0.094919145 | 5.54E-06 | 0.00020727 | 1.99E-07 | 0.018939551 | 0.046612639 | 4.06E-23 | 4.66E-16 | 0.005626196 | 0.365752083 | 0.848843567 | 0.554394177 |
|  | Pattern IV | A=B→A>B→A>B | 0.00016127 | 0.038994984 | 0.073735678 | 0.033207669 | 0.076150375 | 3.24E-05 | 0.239771363 | 0.24737139 | 0.38395132 | 0.960238395 | 1.48E-17 | 3.71E-10 | 0.035876595 |
|  |  | A=B→A<B→A<B | 0.225154263 | 0.000101806 | 3.10E-07 | 7.94E-08 | 6.20E-06 | 0.245512393 | 0.573951208 | 6.96E-18 | 4.79E-16 | 0.258303749 | 0.171187655 | 0.013488826 | 0.224378533 |
|  |  | A>B→A=B→A=B | 1.19E-05 | 2.23E-20 | 4.29E-07 | 8.32E-81 | 6.37E-34 | 4.27E-27 | 0.032971166 | 1.16E-43 | 2.84E-23 | 1.72E-05 | 1.10E-51 | 5.55E-30 | 6.71E-05 |
|  |  | A<B→A=B→A=B | 5.21E-140 | 4.22E-19 | 2.30E-89 | 2.24E-07 | 1.30E-18 | 3.87E-05 | 2.76E-07 | 5.23E-36 | 6.73E-19 | 5.41E-07 | 8.59E-30 | 1.08E-14 | 4.35E-06 |
